# Supplementary material for: Motif-based models accurately predict cell type-specific distal regulatory elements
Source: Nat Commun. 2025 Nov 24;16:10370. doi: 10.1038/s41467-025-65362-2 (PMC12644898; doi:10.1038/s41467-025-65362-2)
Supplement: Supplementary file 1 — Supplementary Information [file 41467_2025_65362_MOESM1_ESM.pdf]

Supplementary Information

Table of Contents

Supplementary Figures 3

Supplementary Figure 1. A bag-of-motif (BOM) pipeline 3

Supplementary Figure 2. BOM models for predicting mouse embryonic enhancers 5

Supplementary Figure 3. Confusion matrix for the multiclass classification of mouse E8.25 CREs . 7

Supplementary Figure 4. Comparison with other models 9

Supplementary Figure 5. Prediction of classical CREs and CREs located at superenhancers 10

Supplementary Figure 6. Prediction performance for multiple motif detection thresholds 11

Supplementary Figure 7. TF binding sites are commonly missed using standard cut-offs for motif identification 12

Supplementary Figure 8. BOM model performance dramatically decreases after random subsampling to reduce total motif counts 13

Supplementary Figure 9. BOM performance decreases if overlapping motifs are removed 14

Supplementary Figure 10. Most predictive TF binding motifs of *A. thaliana* root cell types 15

Supplementary Figure 11. Cross-species prediction of human fetal enhancers 16

Supplementary Figure 12. Cross-species prediction of mouse E8.25 enhancers 17

Supplementary Figure 13. The collection of motifs identifies CREs of similar cell types at similar developmental time points between human and mouse 19

Supplementary Figure 14. Performance metrics for the prediction of human heart cells using the mouse model 20

Supplementary Figure 15. Loss and accuracy across DNABERT training epochs 21

Supplementary Figure 16. Deep learning architectures 22

Supplementary Figure 17. BOM running times across datasets 23

Supplementary Tables 24

Supplementary Table 1. Comparison of tools for the discrimination of motifs underlying regulatory elements 24

Supplementary Table 2. Number of CREs used in BOM models 25

Supplementary Table 3. Summary of performance metrics of binary models 30

Supplementary Table 4. Summary of prediction statistics for random mouse E8.25 data splits 33

Supplementary Table 5. Summary of BOM multiclass test results using different error functions .. 33

Supplementary Table 6. Summary of performance metrics of mouse E8.25 multiclass models 34

Supplementary Table 7. Flanking regions prediction statistics 37

Supplementary Table 8. CRE number subsample prediction statistics 37

Supplementary Table 9. Prediction statistics for mouse E8.25 topics 38

Supplementary Table 10. Summary of prediction statistics of mouse E8.5 CREs using models trained on mouse E8.25 CREs 38

Supplementary Table 11. Super enhancers (SE) vs. non-super enhancer (classical) prediction statistics 38
Supplementary Table 12. Summary of prediction statistics for different motif detection thresholds 38
Supplementary Table 13. Summary of prediction statistics of BOM models trained on overlapping and non-overlapping TF binding motifs counts 39
Supplementary Table 14. Prediction statistics for human cell lines 39
Supplemental Table 15. Summary of prediction statistics of human hematopoiesis enhancers 39
Supplemental Table 16. Summary of prediction statistics of zebrafish enhancers 39
Supplementary Table 17. *A. thaliana* enhancers prediction statistics 39
Supplementary Table 18. Human fetal enhancers prediction statistics 39
Supplementary Table 19. Cross-species prediction statistics of cell type specific CREs 40
Supplementary Table 20. Mouse adult heart enhancers prediction statistics 40
Supplementary Table 21. Human adult heart enhancers prediction statistics 40
Supplementary Table 22. Cross-species prediction of adult heart cell type-specific CREs 40
Supplementary Table 23. Human adult heart cell prediction statistics. 41
Supplementary Table 24. Summary of prediction performance metrics for BOM models trained using different tree depth values 41
Supplementary Table 25. Summary of performance of DNN multiclass models on test data 42

Supplementary Figures

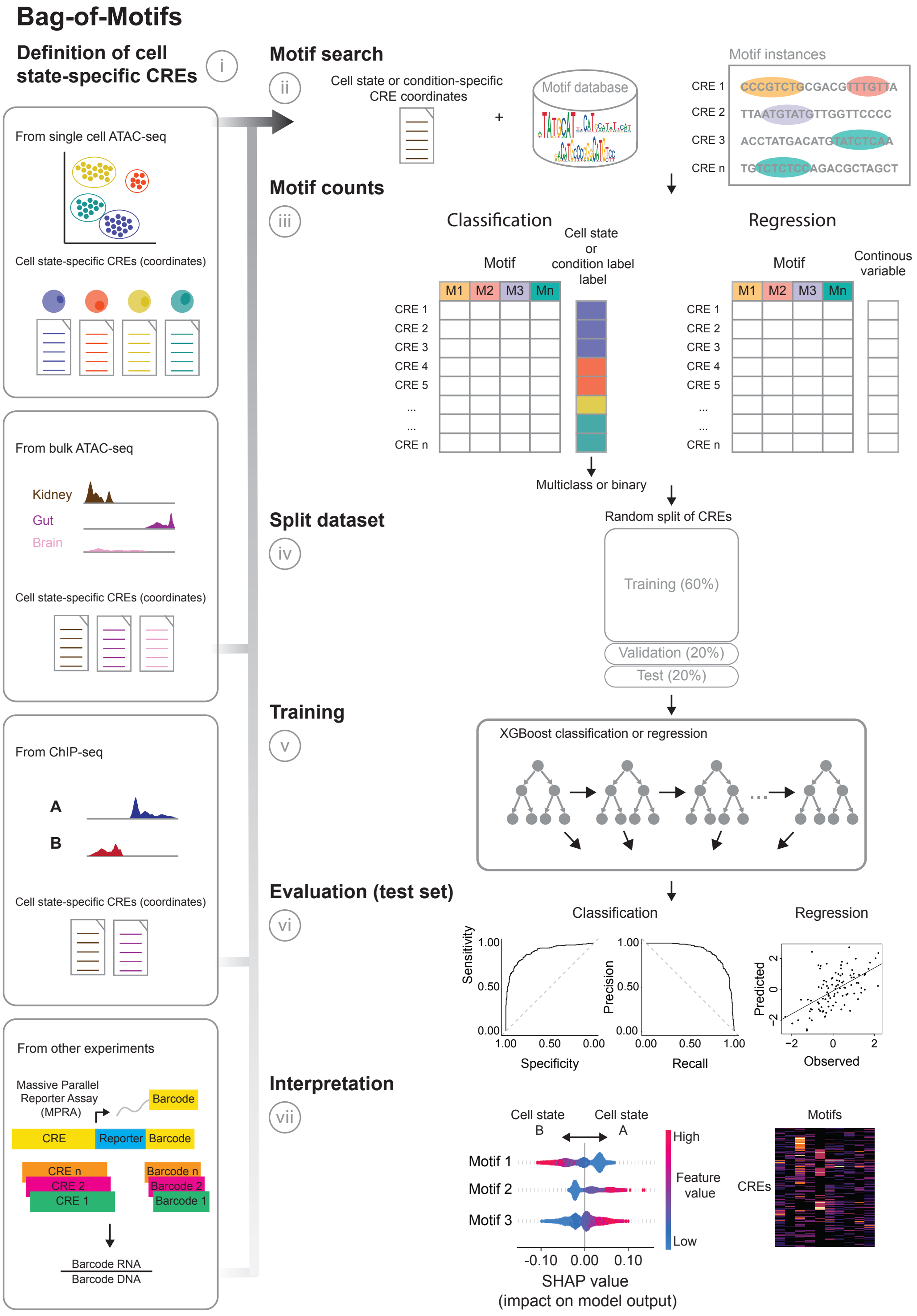

Supplementary Figure 1. A bag-of-motif (BOM) pipeline
The bag-of-motifs is a computational strategy for analyzing cis-regulatory elements (CREs) from different cell contexts. The pipeline can be applied to both classification and regression tasks and involves the following steps:

i. CRE Definition: CREs can be defined using different experimental data, such as single-cell or bulk ATAC-seq, or histone mark profiling (e.g., H3K27ac for active enhancers). These

defined CREs are specific to particular cell states and can be labeled for binary or multiclass classification.

ii. TF Binding Motif Identification: FIMO [1] enumerates TF-binding motif instances within the defined CRE sequences based on motifs from PWM databases.
iii. Motif Frequency Matrix: A matrix is constructed with the frequency of motifs in each CRE. This matrix serves as input for subsequent model training.
iv. Dataset Splitting: The dataset is divided into training, validation, and test sets, with proportions of 60%, 20%, and 20%, respectively.
v. Model Training: An XGBoost [2] model is trained to predict target labels/values. Model performance is assessed during training on the validation set. Training terminates upon reaching a maximum number of iterations or when validation set performance ceases to improve after a specific number of iterations.
vi. Model Evaluation: The trained model's predictive performance is evaluated on the test dataset. Precision-recall curves and Receiver Operating Characteristic (ROC) curves are visualized for classification models. Higher values under the curves indicate better classification performance. For regression models, a correlation measure is calculated to assess prediction performance.
vii. SHAP Value Analysis: SHAP values are calculated to identify the TF binding motifs with the greatest influence on the predicted outcomes for each CRE.

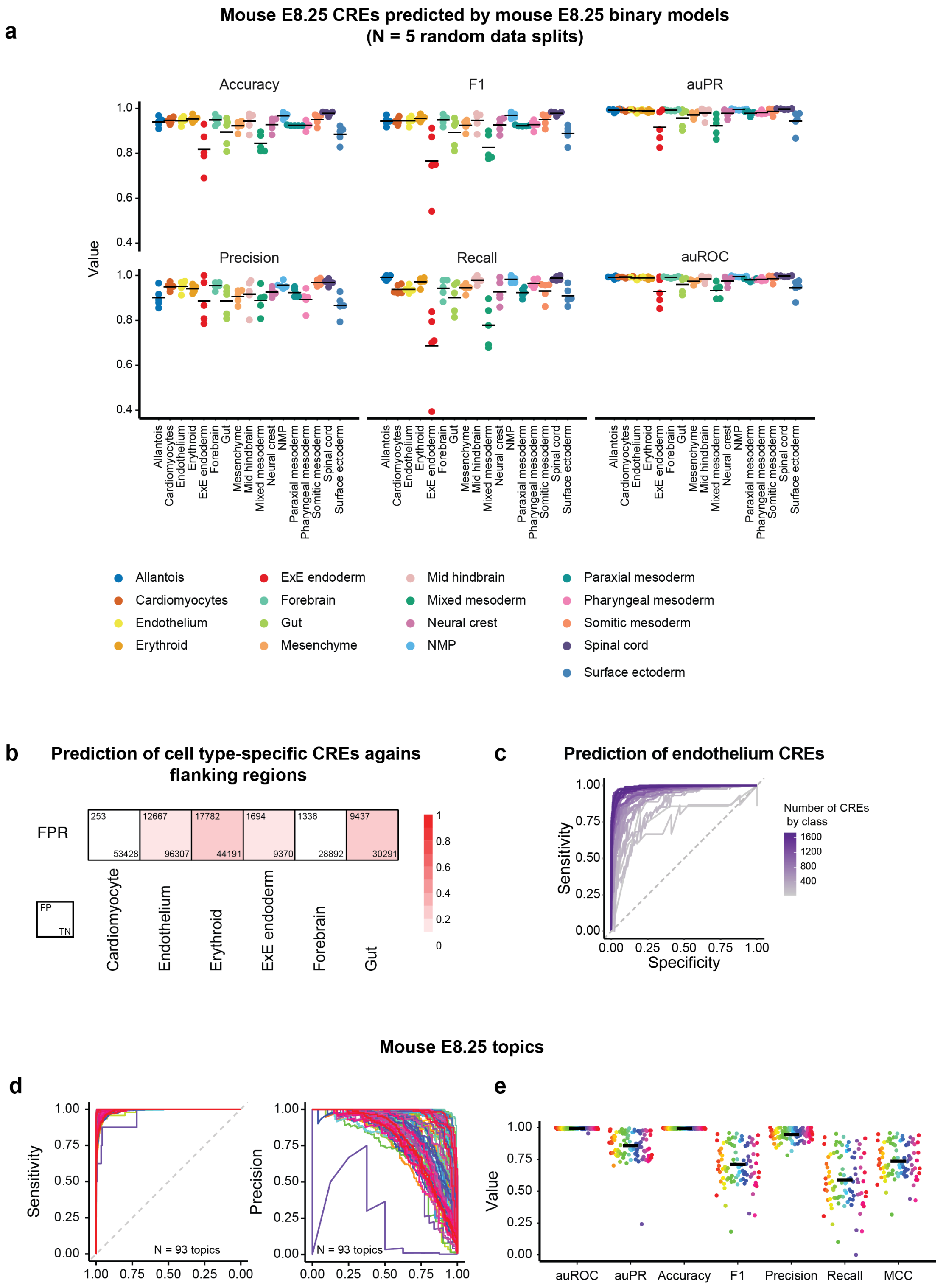

Supplementary Figure 2. BOM models for predicting mouse embryonic enhancers

a. Performance metrics for predicting cell type-specific enhancers in 17 mouse E8.25 cell types using binary BOM models [3]. Accuracy, F1 score, area under the precision-recall curve (auPR), precision, recall, and are under the Receiver Operating Characteristic (ROC) curve (auROC) were calculated across 5 binary BOM models trained with different random data splits. Horizontal bars represent the mean value across the 5 test sets from the random data splits. The colors represent different cell types. **c**. False discovery rate (FDR) for the prediction of cell type-specific enhancers and CRE-flanking regions (**methods**). **c.** ROC curves for the prediction of endothelium CREs against a background of CREs specific to other mouse E8.25 cell types. The colour represents the number of CREs sampled from each class without replacement (positive class = endothelium CREs, negative class = background CREs; n = 35 subsets) (**methods**). **d-e**. Prediction performance of multiclass BOM models trained to

predict 93 mouse E8.25 topics across 15,321 test regions (**Methods**). **d**. ROC (left) and precision-recall (right) curves are shown. **e**. Performance metrics of models trained to predict mouse E8.25 topics. Area under the ROC curve (auROC), area under the precision-recall curve (auPR), accuracy, F1 score, precision, recall and MCC are shown. Horizontal bars indicate the mean value for every statistic. Source Data are provided as a Source Data file.

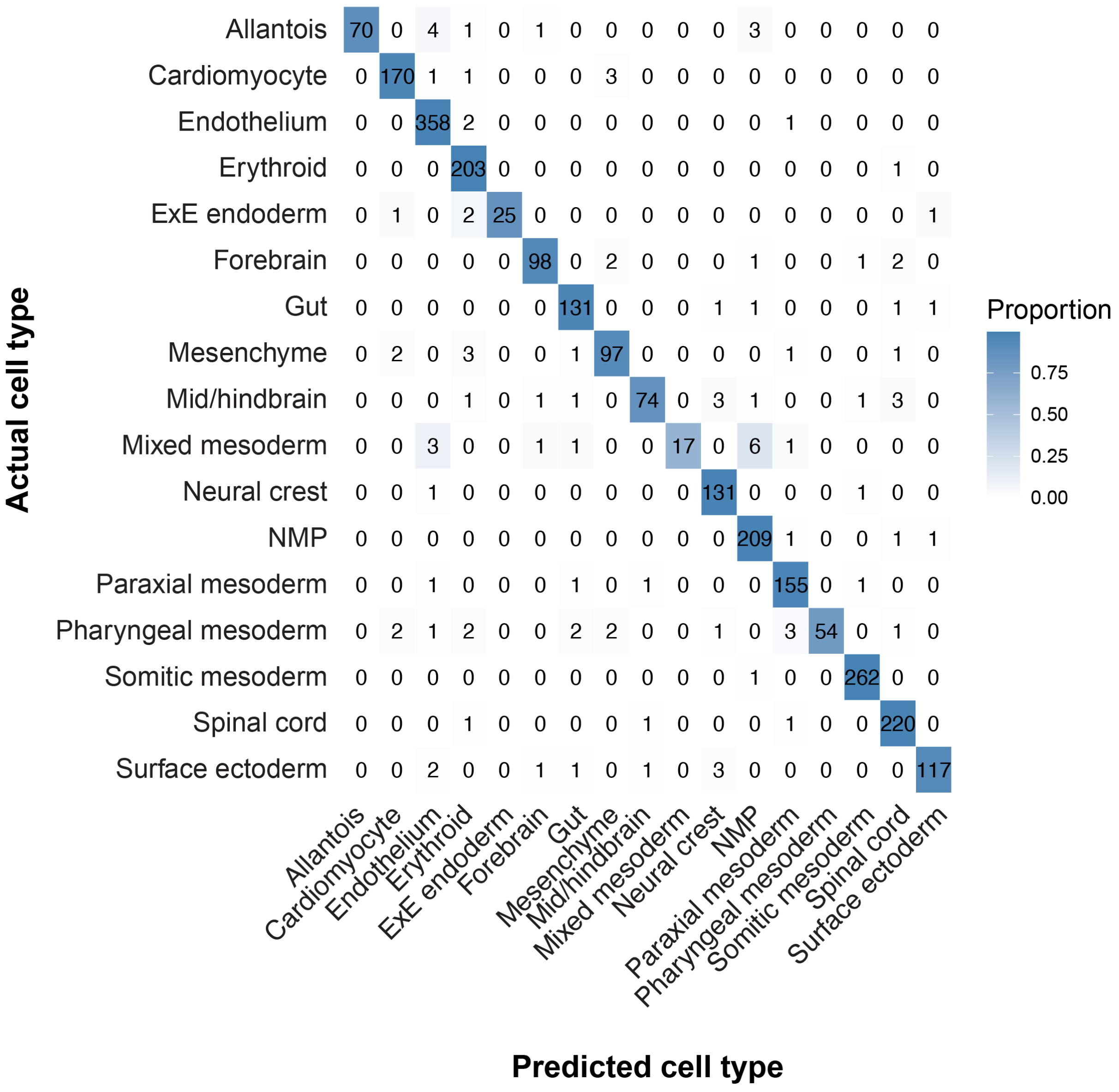

Supplementary Figure 3. Confusion matrix for the multiclass classification of mouse E8.25 CREs
Confusion matrix of mouse e8.25 CREs classified by BOM. Source Data are provided as a Source Data file.

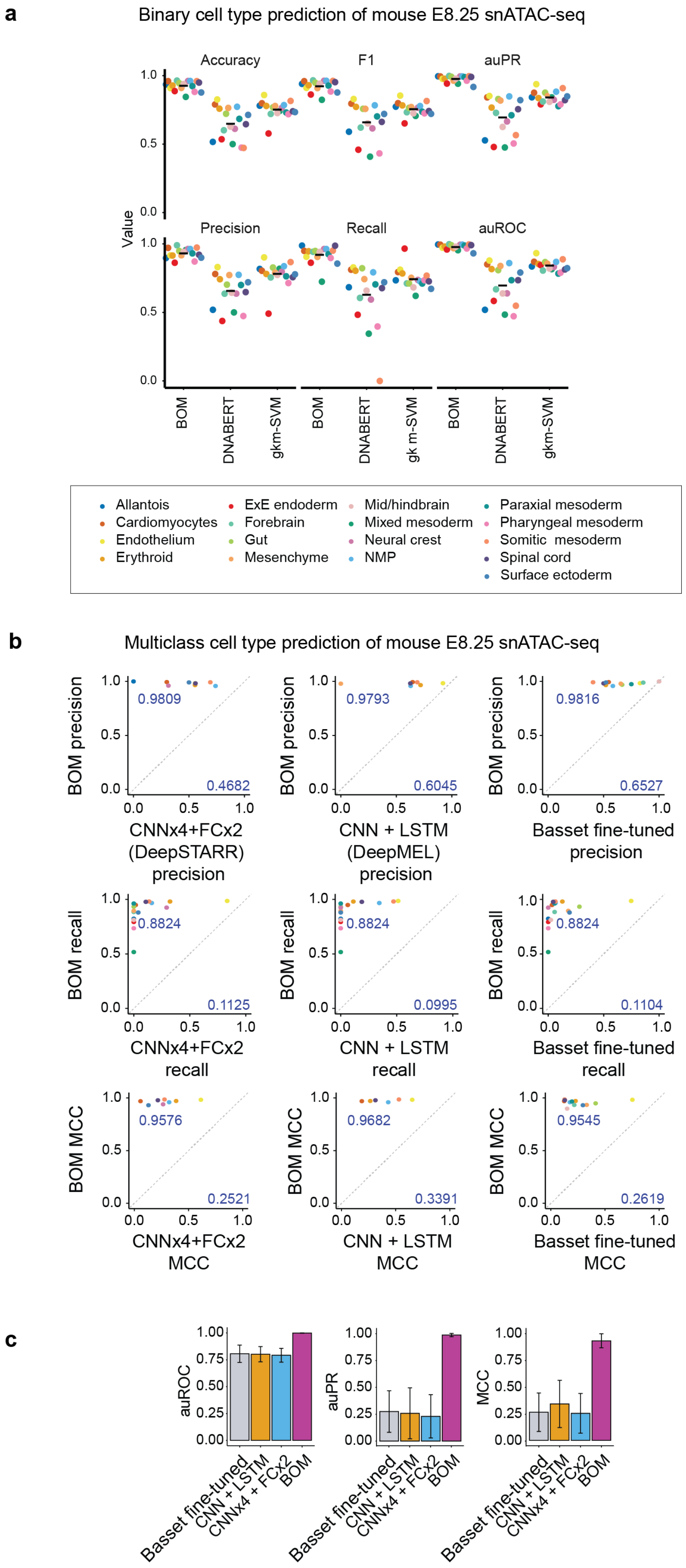
a
Binary cell type prediction of mouse E8.25 snATAC-seq
Accuracy
F1
auPR
Precision
Recall
auROC
Value
1.0
0.5
0.0
BOM
DNABERT
gkm-SVM
Allantois
Cardiomyocytes
Endothelium
Erythroid
ExE endoderm
Forebrain
Gut
Mesenchyme
Mid/hindbrain
Mixed mesoderm
Neural crest
NMP
Paraxial mesoderm
Pharyngeal mesoderm
Somitic mesoderm
Spinal cord
Surface ectoderm
b
Multiclass cell type prediction of mouse E8.25 snATAC-seq
BOM precision
0.9809
0.4682
CNNx4+FCx2 (DeepSTARR) precision
0.9793
0.6045
CNN + LSTM (DeepMEL) precision
0.9816
0.6527
Basset fine-tuned precision
BOM recall
0.8824
0.1125
CNNx4+FCx2 recall
0.8824
0.0995
CNN + LSTM recall
0.8824
0.1104
Basset fine-tuned recall
BOM MCC
0.9576
0.2521
CNNx4+FCx2 MCC
0.9682
0.3391
CNN + LSTM MCC
0.9545
0.2619
Basset fine-tuned MCC
c
auROC
auPR
MCC
1.00
0.75
0.50
0.25
0.00
Basset fine-tuned
CNN + LSTM
CNNx4 + FCx2
BOM

Supplementary Figure 4. Comparison with other models
a. Performance metrics comparison for BOM, DNABERT and LS-GKM binary models trained to predict mouse E8.25 cell type-specific enhancers. Accuracy, F1 score, area under the precision-recall curve (auPR), precision, recall and area under the ROC (auROC) curve values are shown for the 17 cell types. Horizontal bars represent the mean value across the 5 test sets from the random data splits. **b**. Performance metrics for predicting cell type-specific enhancers in 17 mouse E8.25 cell types comparing BOM to deep neural network architectures (DNNs) for multiclass predictions [3]. These are common DNN architectures for sequence models in genomic. **c**. Summary of the prediction performance of multiclass classification of 17 mouse E8.25 cell types across BOM and DNN architectures. Source Data are provided as a Source Data file.

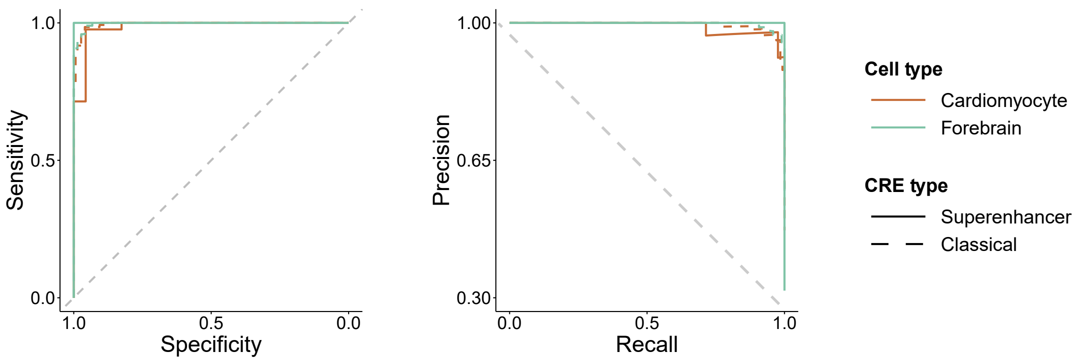

Supplementary Figure 5. Prediction of classical CREs and CREs located at superenhancers

ROC curves (left) and precision-recall curves (right) for the prediction of cell type-specific CREs from background CREs (CRes specific to other cell types). CREs were separated between those located at superenhancers (solid lines; **Methods**) and those not associated to superenhancers ("classical" CREs; dashed lines).

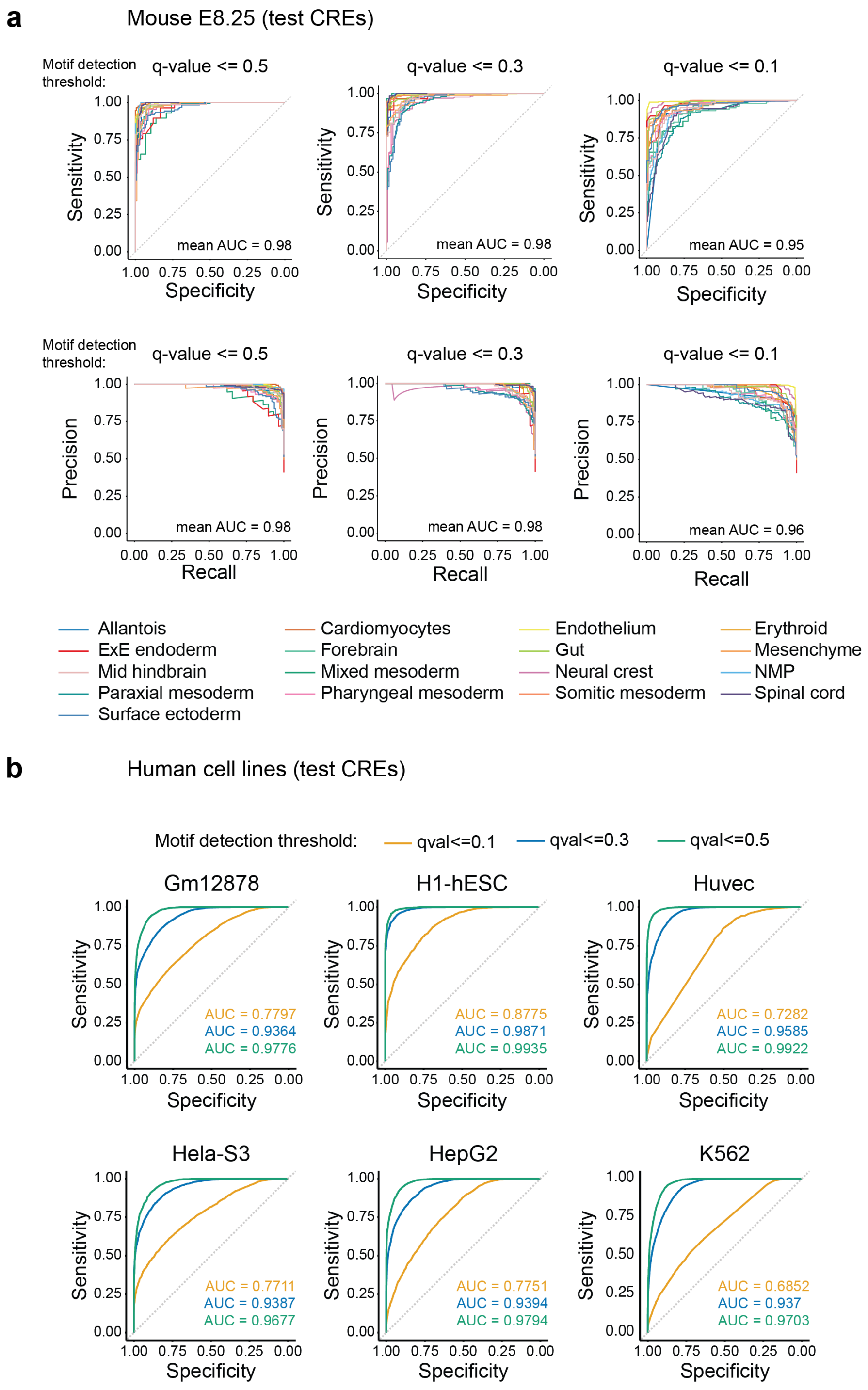

Supplementary Figure 6. Prediction performance for multiple motif detection thresholds
a. Performance of binary BOM models to distinguish mouse E8.25 cell type-specific enhancers across three motif detection thresholds (q-value <= 0.1, q-value <= 0.3, and q-value <= 0.5). Receiver Operating Characteristic (ROC) curves (top) and precision-recall (PR) curves (bottom) are shown. The mean area under the ROC curve (auROC) and area under the precision-recall curve (auPR) are shown for each q-value threshold. **b**. ROC curves are shown for six human cell lines: Gm12878, H1-hESC, Huvec, Hela-S3, HepG2 and K562. ROC curves for three motif detection thresholds are shown (q-value <= 0.1, q-value <= 0.3 and q-value <= 0.5). The corresponding auROC values are shown in each case. Source Data are provided as a Source Data file.

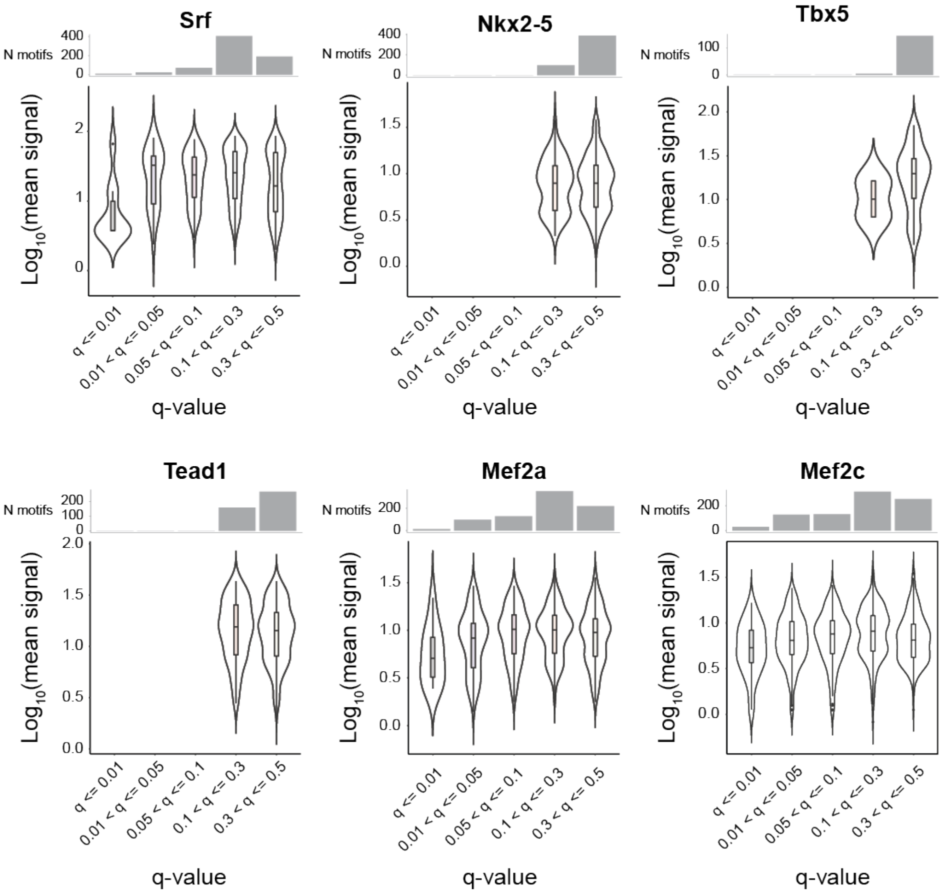

Supplementary Figure 7. TF binding sites are commonly missed using standard cut-offs for motif identification

TF binding signal using bioChIP-seq data compared to motifs of the corresponding TFs identified using FIMO [1] with the Gimme PWMs [4] at mouse embryonic CREs [3] for six cardiac developmental TFs (Mef2a, Mef2c, Nkx2-5, Srf, Tbx5, Tead) [5]. The mean TF binding signal across each TF's ChIP-seq summits are presented on the x-axis. Signals from the two biological replicates per TF were first averaged. TFs were matched using all corresponding motif PWMs. Source Data are provided as a Source Data file.

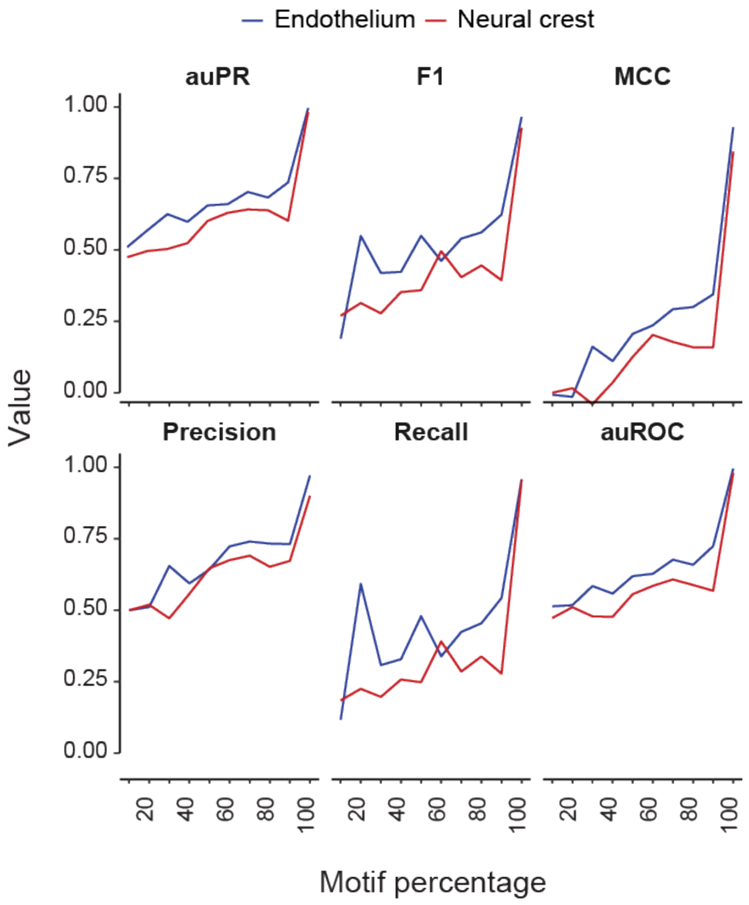

Supplementary Figure 8. BOM model performance dramatically decreases after random subsampling to reduce total motif counts

Performance of models trained for endothelium and neural crest distal CREs in the mouse embryonic test set. Source Data are provided as a Source Data file.

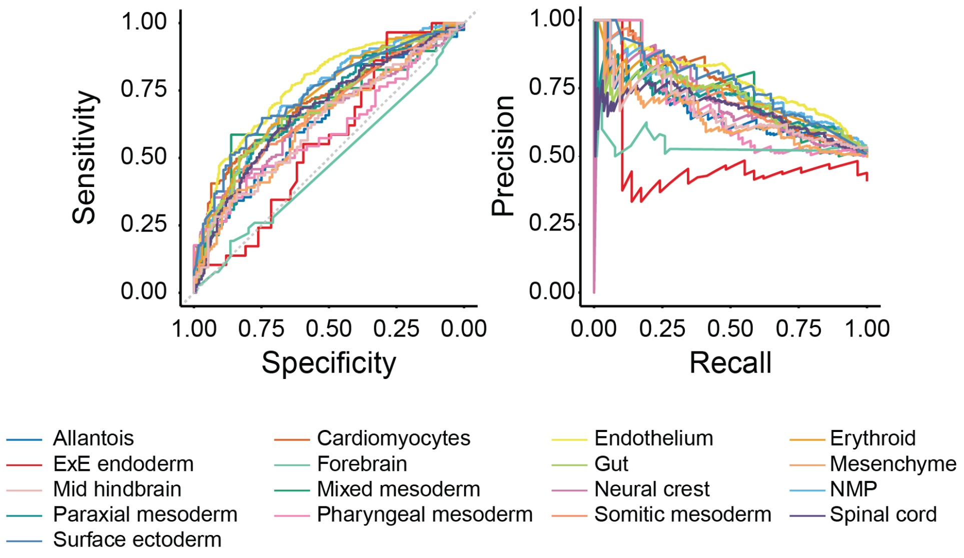

Supplementary Figure 9. BOM performance decreases if overlapping motifs are removed
Performance of models trained on non-overlapping motif counts. Non-overlapping motifs were defined as the ones with best motif scores; **Methods**). Receiver Operating Characteristic (ROC) curves (right) and precision-recall curves (left) are shown. A binary model was trained for each of the 17 mouse E8.25 cell types. The area under the ROC and precision-recall curves are shown in **Supplementary Table 12**. Source Data are provided as a Source Data file.

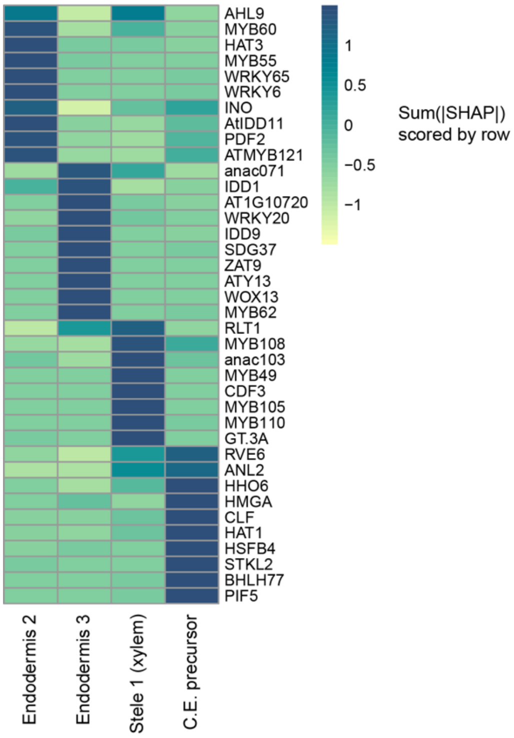

Supplementary Figure 10. Most predictive TF binding motifs of *A. thaliana* root cell types
The heatmap shows TF binding motifs among the top 10 motifs in each cell type (Endodermis 2 and 3, xylem and precursor of cortex and endoderm). Top motifs were selected based on the sum of absolute SHAP values from the BOM multiclass model trained to classify cell type-specific CREs among the four cell types shown (**Methods**). Source Data are provided as a Source Data file.

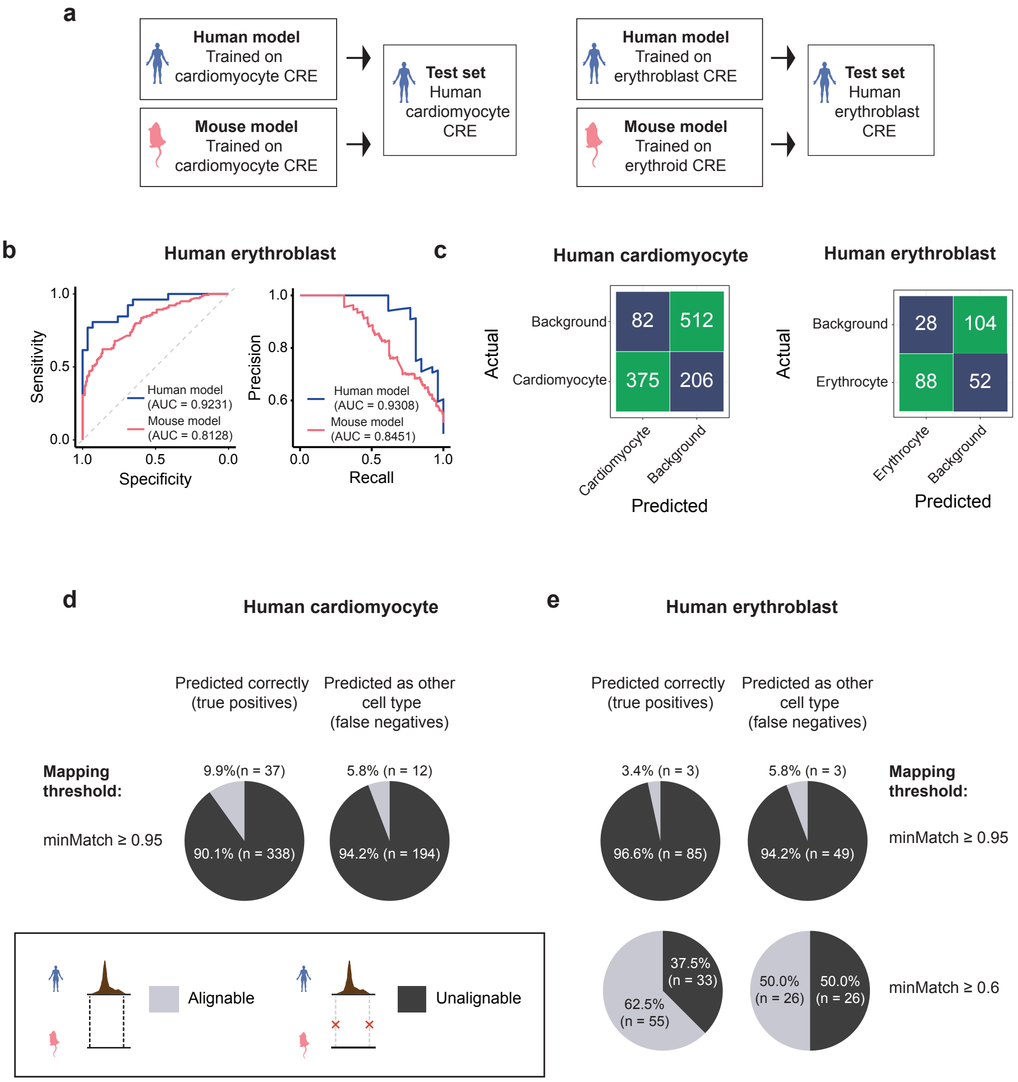

Supplementary Figure 11. Cross-species prediction of human fetal enhancers
a. Binary BOM models were trained to predict human fetal or mouse E8.25 cardiomyocyte or erythroid/erythroblast-specific enhancers. After training, the models trained on both species' data were used to predict human fetal enhancers. **b**. Left: ROC curves for predicting erythroblast-specific enhancers. The ROC curve obtained with the BOM model trained on mouse data is depicted in pink, while the curve for predictions carried out the model trained on human data is shown in blue. The corresponding values of the area under the ROC curves are provided. Right: PR curves for predicting human erythroblast-specific enhancers using models trained on mouse or human data. Curves are colored as in the left panel. Values for the area under the PR curves are shown for each model. **c**. Confusion matrices for predicting human cardiomyocyte (left) or erythroblast (right) specific enhancers using models trained to predict mouse enhancers. **d**. Proportion of human cardiomyocyte-specific enhancers alignable (gray) or unalignable (black) to the mouse genome. Enhancers are categorized as true positives and false negatives. The threshold minMatch >= 0.95 was used in liftOver. **e**. Proportion of erythroblast-specific enhancers alignable (gray) or unalignable (black) to the mouse genome. Like **d**, enhancers are categorized as true positives and false negatives. The top row of pie charts illustrates the proportion of enhancers alignable and not alignable using the default threshold in liftOver (minMatch >= 0.95). The bottom row shows the proportions using the threshold minMatch >= 0.6. Source Data are provided as a Source Data file.

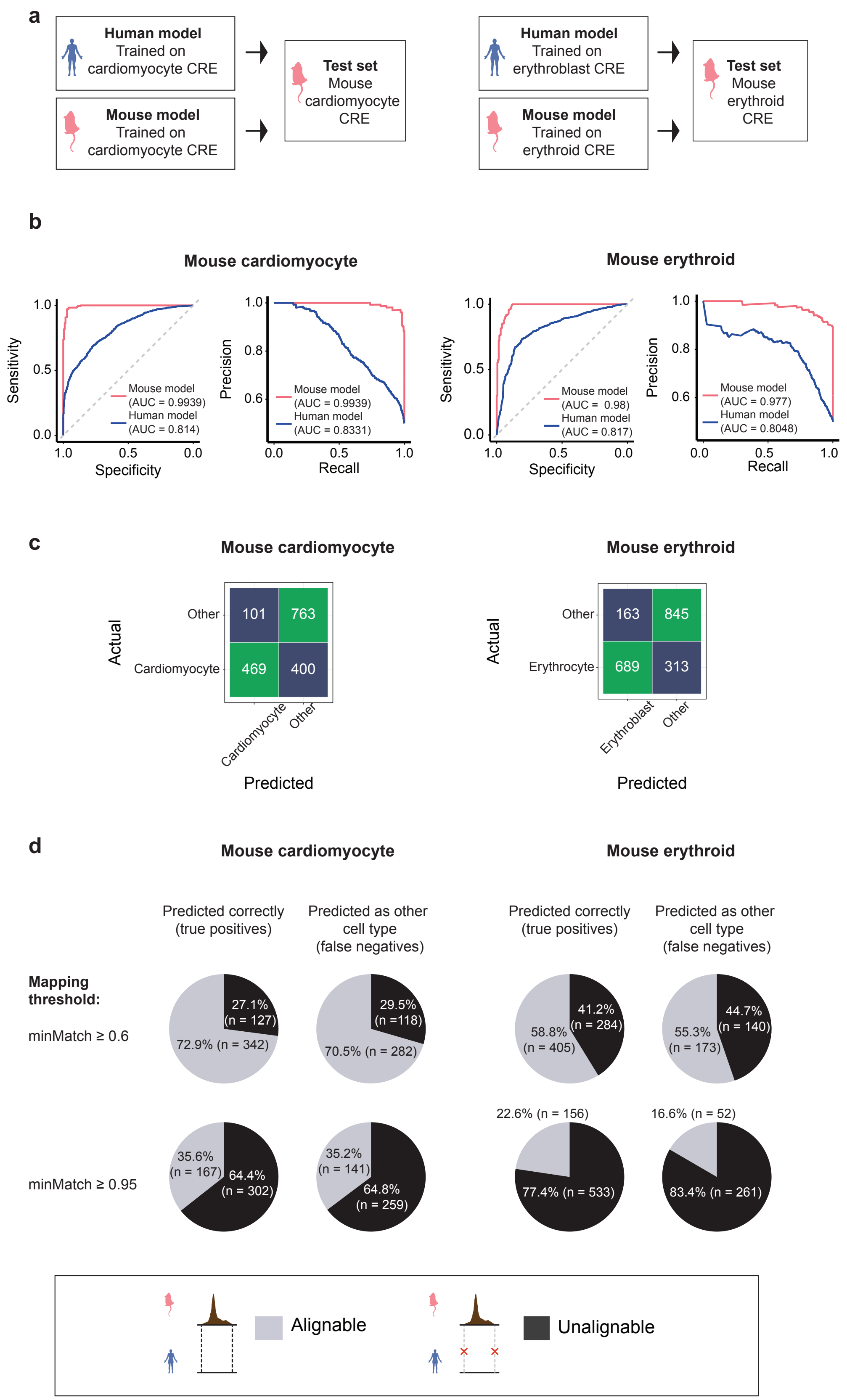

Supplementary Figure 12. Cross-species prediction of mouse E8.25 enhancers

a. Binary BOM models were trained to predict human fetal or mouse E8.25 cardiomyocyte or erythroid/erythroblast-specific enhancers. After training, the models trained on both species' data were

utilized to predict mouse E8.25 enhancers. **b**. Top row: ROC curves for predicting cardiomyocyte (left) or erythroid (right) specific enhancers. The ROC curves obtained with BOM models trained on mouse data are depicted in pink, while the curves for predictions carried out with models trained on human data are shown in blue. The corresponding values of the area under the ROC curves are provided. Bottom row: PR curves for predicting mouse cardiomyocyte (left) or erythroid (right) specific enhancers using models trained on mouse or human data. **c**. Confusion matrices for predicting mouse cardiomyocyte (left) or erythroid (right) specific enhancers using models trained to predict human enhancers. **d**. Proportion of mouse cardiomyocyte (left) and erythroid (right) specific enhancers alignable (gray) or unalignable (black) to the human genome. Enhancers are categorized as true positives and false negatives. The top row of pie charts illustrates the proportion of enhancers alignable and not alignable using the threshold minMatch >= 0.6 in liftOver. The bottom row shows the proportions using the default threshold (minMatch >= 0.95). Source Data are provided as a Source Data file.

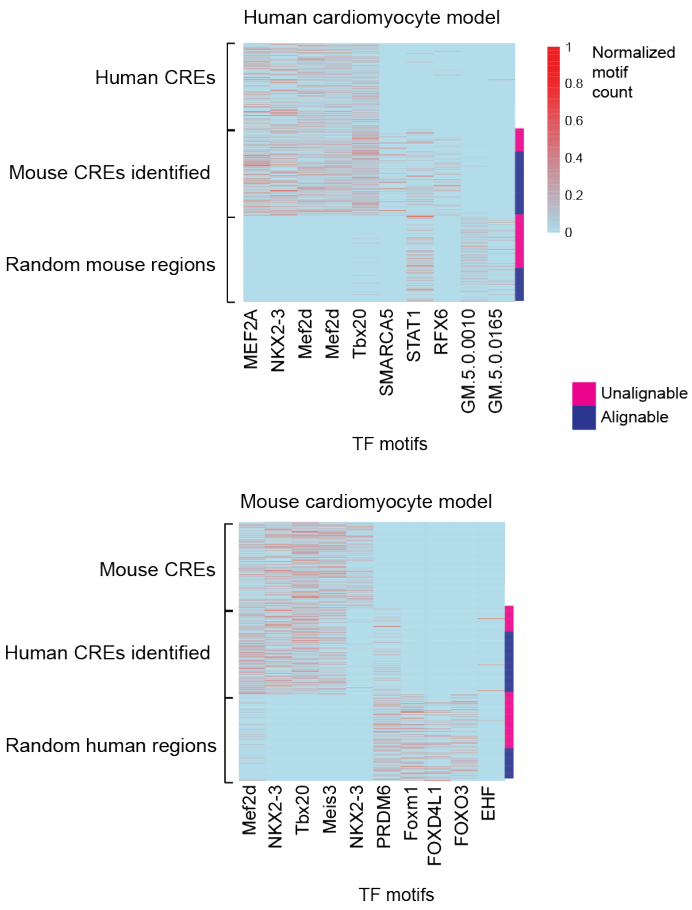

Supplementary Figure 13. The collection of motifs identifies CREs of similar cell types at similar developmental time points between human and mouse

Motif counts for human and mouse cell-type specific CREs for cardiomyocytes are compared to counts of sequences from the genome background. Human cardiomyocyte-specific CREs used to test the model trained using human cardiomyocytes are shown as the first rows in the top heatmap. Motif counts for accurately predicted mouse CREs are shown in the following rows. Random regions in the mouse genome of similar lengths are displayed in the bottom rows of the same heatmap. Motifs were chosen based on their ranked predictive values in predicting the positive cardiomyocyte class (top five motifs in columns 1-5) and the negative background (top five motifs in columns 6-10). The bottom heatmap shows the analogous information for a model trained on the mouse cardiomyocyte-specific CREs. Source Data are provided as a Source Data file.

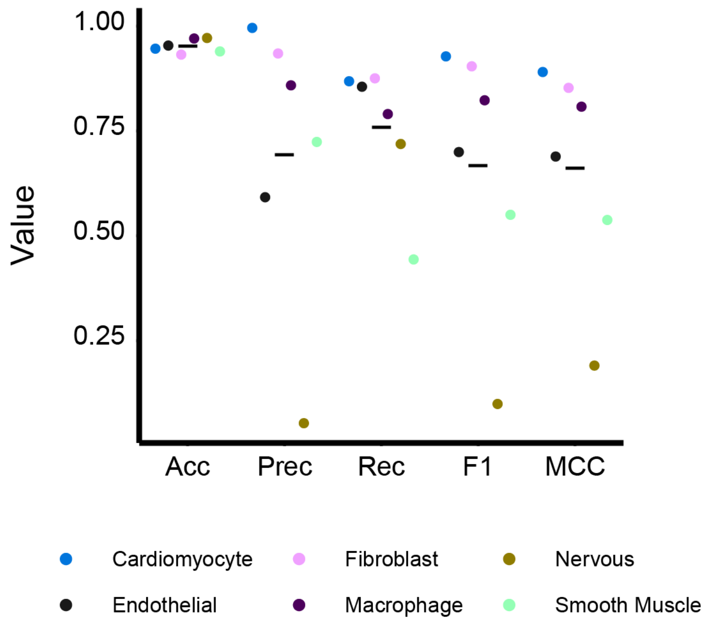

Supplementary Figure 14. Performance metrics for the prediction of human heart cells using the mouse model
Accuracy (Acc), precision (Prec), recall (Rec), F1 scores, and Mattews correlation coefficient (MCC) values are shown for each cell type. Only common cell types between the two datasets are shown. Source Data are provided as a Source Data file.

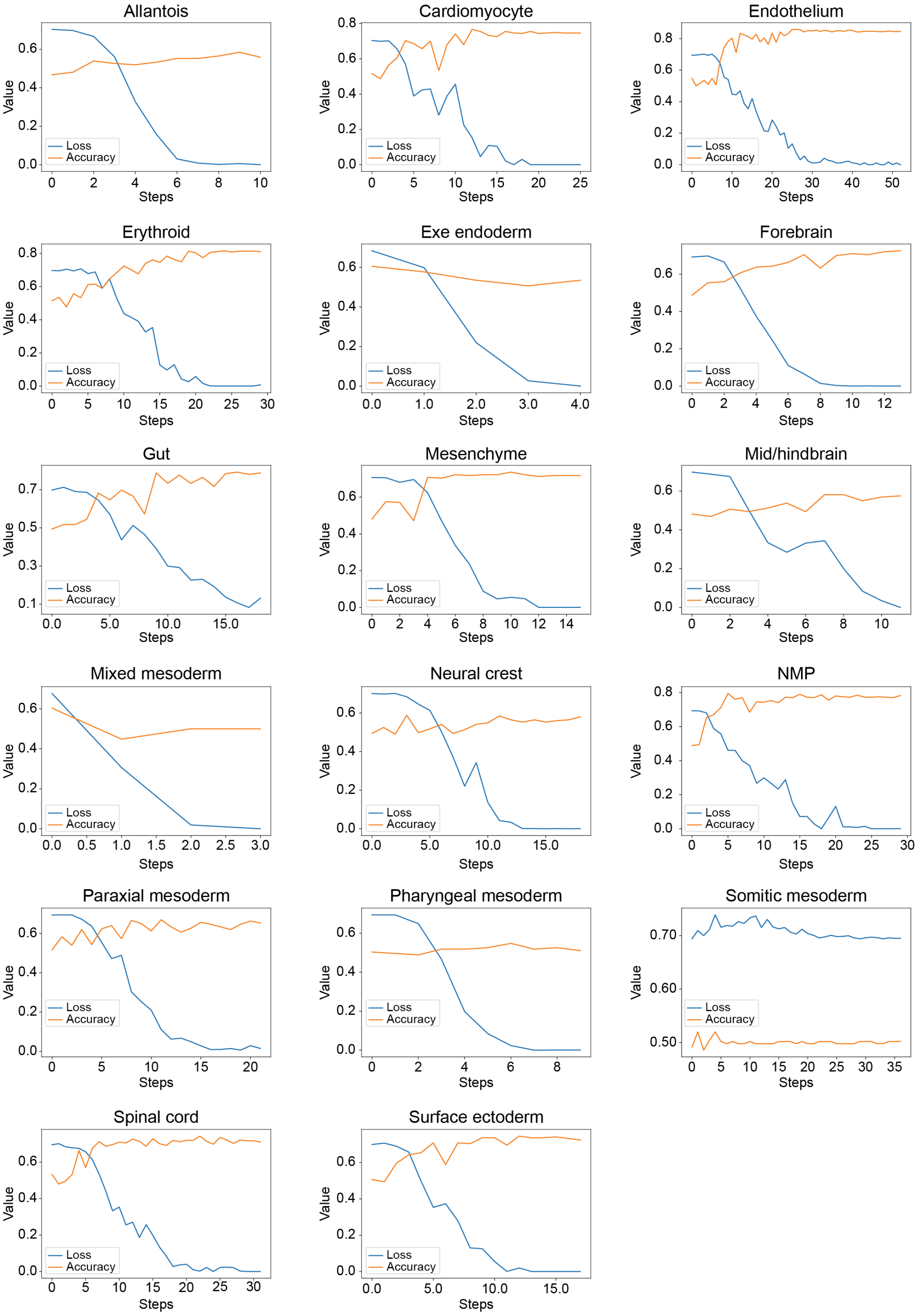

Supplementary Figure 15. Loss and accuracy across DNABERT training epochs
DNABERT models were trained to distinguish CREs specific to a cell type against a background set for each of the mouse E8.25 cell types. The loss and accuracy calculated on the validation set are shown across steps for each model.

Basset

- Input × ∈ {0, 1}500×4 (one-hot encoding of 500 nt sequence)
- 1D convolution (300 filters, size 19, stride 1)
- BatchNorm + ReLU
- Max-pooling (size 3, stride 3)
- 1D convolution (200 filters, size 11, stride 1)
- BatchNorm + ReLU
- Max-pooling (size 4, stride 4)
- 1D convolution (200 filters, size 7, stride 1)
- BatchNorm + ReLU
- Max-pooling (size 4, stride 4)
- Fully-connected (1000 units)
- BatchNorm + ReLU
- Dropout(0.3)
- Fully-connected (1000 units)
- BatchNorm + ReLU
- Dropout(0.3)
- Fully-connected output (17 units, Sigmoid)

DeepMEL

- Input × ∈ {0, 1}500×4 (one-hot encoding of 500 nt sequence)
- 1D convolution (128 filters, size 20, stride 1, ReLU)
- Max-pooling (size 10, stride 10)
- Dropout (0.2)
- TimeDistributed (Dense (128 units, ReLU))
- Bidirectional (LSTM(128 units))
- Dropout(0.2)
- Fully-connected (256 units, ReLU)
- Dropout(0.4)
- Fully-connected output (17 units, Sigmoid)

DeepSTARR

- Input × ∈ {0, 1}500×4 (one-hot encoding of 500 nt sequence)
- 1D convolution (256 filters, size 7, stride 1)
- BatchNorm + ReLU
- Max-pooling (size 2, stride 2)
- 1D convolution (60 filters, size 3, stride 1)
- BatchNorm + ReLU
- Max-pooling (size 2, stride 2)
- 1D convolution (60 filters, size 5, stride 1)
- BatchNorm + ReLU
- Max-pooling (size 2, stride 2)
- 1D convolution (120 filters, size 3, stride 1)
- BatchNorm + ReLU
- Max-pooling (size 2, stride 2)
- Fully-connected (256 units)
- BatchNorm + ReLU
- Dropout(0.4)
- Fully-connected (1000 units)
- BatchNorm + ReLU
- Dropout(0.4)
- Fully-connected output (17 units, Sigmoid)

Supplementary Figure 16. Deep learning architectures
Three deep learning architectures are shown as used in Basset (CNNx3 + FCx2), DeepSTARR (CNNx4 + FCx2) and DeepMEL (CNN + LSTM).

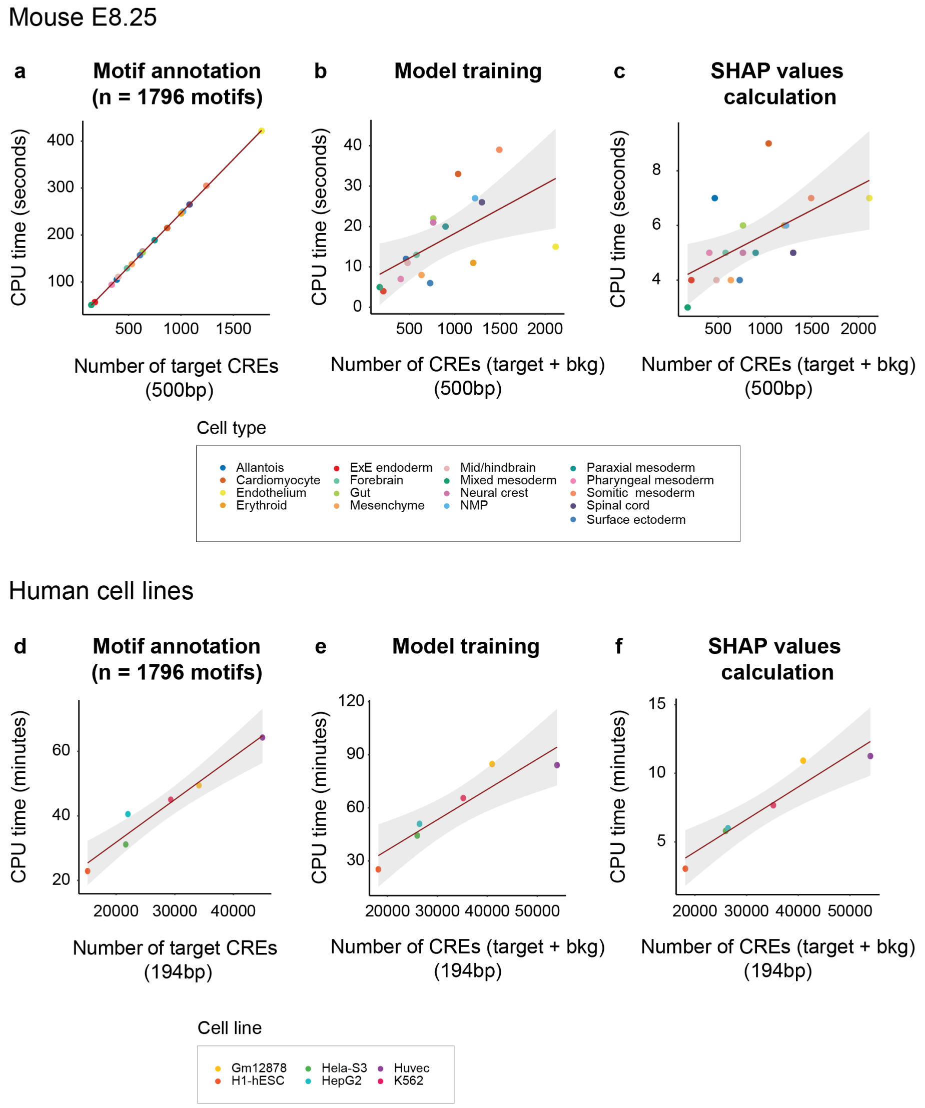

Supplementary Figure 17. BOM running times across datasets
(a-c) Processing time of motif annotation **(a)**, model training **(b)** and SHAP score calculation **(c)** for mouse E8.25 CREs [3]. Motif annotation was carried out using a single CPU while model training and SHAP score calculation were executed in 4 CPUs. The x-axis in **(a)** represents the number of cell-type-specific CREs per cell type (different colors represent different cell types as indicated in the box). In **(b)** and **(c)**, the x-axis the number of training instances (target and background) in each subset per cell type. Best fit line across the data points is shown in red. Shaded region represents the 95% confidence interval of the line of best fit. **(d-f)** Similar to **(a-c)** for human cell lines [6]. The CPU time is shown in seconds for mouse CREs and in minutes for human CREs. Source Data are provided as a Source Data file.

Supplementary Tables

Supplementary Table 1. Comparison of tools for the discrimination of motifs underlying regulatory elements

Method	Modeling approach	Input requirements	Global interpretability	Single-CRE interpretability	Single nucleotide interpretability	Incorporation of distal CREs	Performance metrics	Identification of important TF motifs	Allows more than two samples or classes
**BOM**	Gradient boosting (XGBoost)	CRE coordinates/sequences	✓	✓	✗	✓	✓	✓	✓
**IMAGE** [7]	Regularized linear model (Ridge regression)	CRE coordinates	✓	✗	✗	✓	✗	✓	✗
**ISMARA** [8]	Linear model	Genome-wide gene expression or chromatin state data	✓	✗	✗	✗	✓	✓	✓
**lsGKM** [9]	Support Vector Machines	CRE sequences	✓	✓	✓ With DeltaSVM	✓	✓	✓	✗
**ChromVAR** [10]	Bias-corrected accessibility deviation scores	Chromatin accessibility profile(s) (aligned reads), chromatin accessibility peaks, PWMs or genomic annotations.	✓	✗	✗	✓	✗	✓	✓
**Gimme maelstrom** [4]	**Classification tasks:** Hypergeometric test, Mann–Whitney *U* test, random forest, multiclass classifier using block coordinate descent **Regression tasks:** Ridge regression, regression using boosted trees (XGBoost), multiclass regression, LASSO regression.	CRE coordinates	✓	✗	✗	✓	✗	✓	✗

Supplementary Table 2. Number of CREs used in BOM models

Number of cell types, cell lines, and condition-specific CREs used in BOM models. The numbers of training, validation, and test CREs are provided. The type of BOM model is indicated (binary, multiclass or regression). For binary models, the number of CREs represents the number of CREs for the target cell type of condition. For the multiclass model, the number of CREs represents the contribution from every "class" (e.g., cell type) to the dataset.

Dataset / Model type	Cell type (condition)	N CREs (training)	N CREs (validation)	N CREs (test)	total
Mouse (E8.25) / Binary /multiclass	Allantois	235	74	79	388
	Erythroid	606	192	204	1002
	ExE endoderm	109	41	29	179
	Gut	371	129	135	635
	mesenchyme	314	112	105	531
	NMP	608	200	212	1020
	Paraxial mesoderm	445	145	159	749
	Pharyngeal mesoderm	205	66	68	339
	Somitic mesoderm	731	248	263	1242
	Spinal cord	652	207	223	1082
	Surface ectoderm	361	123	125	609
	Cardiomyocyte	516	178	175	869
	Endothelium	1059	348	361	1768
	Forebrain	273	108	104	485
	Mid/hindbrain	234	83	85	402
	Neural crest	372	129	133	634
	Mixed mesoderm	82	34	29	145
Fruit fly S2 cells / Regression	Housekeeping and developmental enhancer activity	3772	1257	1258	6287
Zebrafish (adult) / Binary	Blood	7896	2582	2627	13105
	Intestine	5931	1964	2018	9913
	Skin	18763	6258	6216	31237
	Brain	12514	4182	4180	20876
	Kidney	7311	2393	2439	12143
	Spleen	3732	1278	1276	6286
	Colon	2997	997	1008	5002
	liver	5084	1652	1690	8426
	testis	12505	4170	4173	20848
	heart	8301	2736	2747	13784
	muscle	4366	1429	1453	7248
Human (fetal) / Binary	Cardiomyocyte	344	117	120	581
	Erythroblasts	82	32	26	140
Human (cell lines) / Binary	Gm12878	20483	6862	6776	34121
	H1-hESC	9158	2974	3001	15133
	Hela-S3	12998	4349	4284	21631
	HepG2	13196	4428	4377	22001
	Huvec	26926	9122	8903	44951
	K562	17629	5883	5799	29311
Human (hematopoiesis) / Binary	B	484	159	168	811
	CD14 monocyte 1	508	170	173	851
	CD14 monocyte 2	296	110	101	507

Dataset / Model type	Cell type (condition)	N CREs (training)	N CREs (validation)	N CREs (test)	total
	CD4 M	85	33	25	143
	CD4 N2	70	23	26	119
	CD8.CM	228	77	82	387
	CD8.EM	182	59	64	305
	cDC	334	109	116	559
	CLP 1	392	134	143	669
	CLP 2	508	170	173	851
	CMP/LMPP	295	107	100	502
	Early basophil	508	170	173	851
	Early erythroid	508	170	173	851
	GMP	508	170	173	851
	GMP/Neutrophil	508	170	173	851
	HSC	135	47	44	226
	Late erythroid	64	22	17	103
	NK	420	141	153	714
	pDC	508	170	173	851
	Plasma	243	87	90	420
	Pre B	508	170	173	851
	Unknown 26	310	110	103	523
**Human acute myeloid leukemia (AML) / Binary**	AML	6632	2200	2214	11046
	Healthy	6623	2218	2205	11046
**Mouse topics (E8.25) / Multiclass**	Topic2	741	243	237	1221
	Topic3	734	240	245	1219
	Topic4	56	23	20	99
	Topic6	223	63	65	351
	Topic7	377	120	137	634
	Topic8	255	110	113	478
	Topic9	186	71	87	344
	Topic10	181	78	69	328
	Topic11	684	192	212	1088
	Topic12	614	208	221	1043
	Topic14	371	137	115	623
	Topic16	422	151	147	720
	Topic17	269	105	98	472
	Topic18	567	162	188	917
	Topic19	548	177	192	917
	Topic20	364	152	139	655
	Topic21	513	165	141	819
	Topic22	235	83	63	381
	Topic23	812	240	266	1318

Dataset / Model type	Cell type (condition)	N CREs (training)	N CREs (validation)	N CREs (test)	total
	Topic24	712	235	246	1193
	Topic25	575	173	158	906
	Topic26	503	161	201	865
	Topic27	307	96	104	507
	Topic28	782	248	282	1312
	Topic29	153	53	46	252
	Topic30	621	191	200	1012
	Topic31	313	128	120	561
	Topic32	760	271	239	1270
	Topic33	392	110	112	614
	Topic34	673	225	251	1149
	Topic35	491	176	163	830
	Topic36	754	251	247	1252
	Topic37	590	164	162	916
	Topic38	337	110	100	547
	Topic39	760	267	271	1298
	Topic40	186	78	57	321
	Topic41	445	132	147	724
	Topic42	383	105	136	624
	Topic43	491	158	172	821
	Topic44	577	203	197	977
	Topic45	756	237	251	1244
	Topic46	269	89	81	439
	Topic47	259	84	92	435
	Topic48	416	141	146	703
	Topic49	148	50	46	244
	Topic50	616	203	206	1025
	Topic51	439	145	153	737
	Topic52	569	179	214	962
	Topic53	315	121	107	543
	Topic54	740	255	255	1250
	Topic55	648	212	198	1058
	Topic56	773	253	236	1262
	Topic57	358	143	129	630
	Topic58	499	151	175	825
	Topic59	407	158	140	705
	Topic60	727	260	234	1221
	Topic61	439	144	140	723
	Topic62	429	149	127	705
	Topic63	474	178	158	810

Dataset / Model type	Cell type (condition)	N CREs (training)	N CREs (validation)	N CREs (test)	total
	Topic64	687	207	237	1131
	Topic65	658	223	216	1097
	Topic66	779	291	286	1356
	Topic68	524	155	191	870
	Topic69	683	261	228	1172
	Topic70	783	252	231	1266
	Topic71	484	160	165	809
	Topic72	372	130	144	646
	Topic73	284	99	114	497
	Topic74	398	146	113	657
	Topic75	837	277	271	1385
	Topic76	846	278	306	1430
	Topic77	31	13	8	52
	Topic78	108	34	31	173
	Topic79	620	206	185	1011
	Topic80	766	246	243	1255
	Topic81	702	218	204	1124
	Topic82	411	149	138	698
	Topic83	713	230	241	1184
	Topic84	355	111	115	581
	Topic85	258	99	84	441
	Topic86	283	107	86	476
	Topic87	282	85	102	469
	Topic88	640	223	206	1069
	Topic91	566	176	156	898
	Topic92	661	224	234	1119
	Topic93	539	162	147	848
	Topic94	487	144	152	783
	Topic95	383	123	115	621
	Topic96	333	102	102	537
	Topic97	571	190	190	951
	Topic98	663	211	231	1105
	Topic99	623	236	256	1115
	Topic100	394	146	139	679
***A. thaliana* root / Multiclass**	Endodermis 2	79	25	23	127
	Endodermis 3	74	24	29	127
	Xylem (Stele 1)	84	30	25	139
	Precursor of cortex/endodermis	128	43	45	216
**Mouse adult heart**	Cardiomyocyte	5870	1977	1911	9758
	Endothelial	1631	530	515	2676

Dataset / Model type	Cell type (condition)	N CREs (training)	N CREs (validation)	N CREs (test)	total
**/ Multiclass**	Fibroblasts	2316	775	791	3882
	Lymphocyte	808	269	273	1350
	Macrophage	510	163	187	860
	Nervous	402	130	158	690
	Smooth Muscle	253	86	95	434
**Human adult heart / Multiclass**	Cardiomyocyte	3412	1108	1146	5666
	Endothelial	902	333	321	1556
	Fibroblasts	2061	701	645	3407
	Lymphocyte	82	33	16	131
	Macrophage	1656	541	545	2742
	Nervous	262	79	99	440
	Smooth Muscle	1259	417	440	2116

Supplementary Table 3. Summary of performance metrics of binary models
BOM, DNABERT (fine-tuned), LS-GKM, Enformer (fine-tuned) models trained to predict mouse E8.25 distal CREs [3]. Accuracy, F1 score, auPR, precision, recall and auROC were calculated across each of the 17 cell types in the dataset.

Cell type	Model	Accuracy	F1	auPR	Precision	Recall	auROC	MCC
**Allantois**	**BOM**	**0.936**	**0.940**	**0.995**	**0.897**	**0.987**	**0.995**	**0.905**
	DNABERT	0.516	0.590	0.527	0.519	0.684	0.519	0.027
	LS-GKM	0.781	0.773	0.842	0.817	0.734	0.835	0.565
	Enformer	0.748	0.761	0.837	0.738	0.785	0.829	0.497
**Cardiomyocyte**	**BOM**	**0.960**	**0.960**	**0.994**	**0.971**	**0.949**	**0.994**	**0.969**
	DNABERT	0.790	0.796	0.841	0.780	0.811	0.851	0.579
	LS-GKM	0.798	0.799	0.875	0.804	0.794	0.869	0.597
	Enformer	0.795	0.791	0.890	0.817	0.766	0.889	0.592
**Endothelium**	**BOM**	**0.909**	**0.911**	**0.977**	**0.916**	**0.906**	**0.972**	**0.982**
	DNABERT	0.826	0.829	0.851	0.831	0.828	0.879	0.651
	LS-GKM	0.858	0.854	0.938	0.902	0.812	0.932	0.721
	Enformer	0.942	0.943	0.979	0.947	0.939	0.979	0.884
**Erythroid**	**BOM**	**0.928**	**0.930**	**0.977**	**0.915**	**0.946**	**0.980**	**0.971**
	DNABERT	0.759	0.772	0.767	0.742	0.804	0.816	0.519
	LS-GKM	0.779	0.782	0.836	0.781	0.784	0.853	0.557
	Enformer	0.918	0.920	0.978	0.909	0.931	0.979	0.836
**ExE endoderm**	**BOM**	**0.887**	**0.862**	**0.942**	**0.862**	**0.862**	**0.959**	**0.890**
	DNABERT	0.535	0.459	0.479	0.438	0.483	0.584	0.054
	LS-GKM	0.578	0.651	0.790	0.491	0.966	0.845	0.340
	Enformer	0.859	0.853	0.961	0.744	1.000	0.977	0.753
**Forebrain**	**BOM**	**0.964**	**0.966**	**0.998**	**0.990**	**0.942**	**0.997**	**0.933**
	DNABERT	0.601	0.621	0.728	0.636	0.606	0.669	0.201
	LS-GKM	0.720	0.733	0.811	0.755	0.712	0.805	0.441

Cell type	Model	Accuracy	F1	auPR	Precision	Recall	auROC	MCC
	Enformer	0.819	0.847	0.875	0.776	0.933	0.882	0.645
**Gut**	**BOM**	**0.945**	**0.948**	**0.992**	**0.948**	**0.948**	**0.990**	**0.948**
	DNABERT	0.722	0.758	0.829	0.703	0.822	0.809	0.443
	LS-GKM	0.769	0.765	0.876	0.828	0.711	0.867	0.546
	Enformer	0.851	0.866	0.951	0.826	0.911	0.945	0.703
**Mesenchyme**	**BOM**	**0.910**	**0.909**	**0.963**	**0.914**	**0.905**	**0.965**	**0.934**
	DNABERT	0.764	0.757	0.818	0.772	0.743	0.839	0.528
	LS-GKM	0.778	0.771	0.854	0.790	0.752	0.856	0.557
	Enformer	0.859	0.859	0.924	0.851	0.867	0.93	0.717
**Mid/hindbrain**	**BOM**	**0.938**	**0.941**	**0.978**	**0.941**	**0.941**	**0.979**	**0.900**
	DNABERT	0.627	0.651	0.625	0.644	0.659	0.638	0.251
	LS-GKM	0.727	0.725	0.809	0.773	0.682	0.820	0.459
	Enformer	0.814	0.833	0.888	0.790	0.882	0.889	0.629
**Mixed mesoderm**	**BOM**	**0.845**	**0.824**	**0.941**	**0.955**	**0.724**	**0.954**	**0.717**
	DNABERT	0.500	0.408	0.476	0.500	0.345	0.484	0.000
	LS-GKM	0.741	0.706	0.842	0.818	0.621	0.816	0.498
	Enformer	0.810	0.807	0.881	0.821	0.793	0.889	0.621
**Neural crest**	**BOM**	**0.961**	**0.962**	**0.995**	**0.962**	**0.962**	**0.993**	**0.940**
	DNABERT	0.612	0.615	0.665	0.637	0.594	0.639	0.225
	LS-GKM	0.765	0.766	0.824	0.797	0.737	0.832	0.532
	Enformer	0.824	0.844	0.921	0.782	0.917	0.914	0.655
**NMP**	**BOM**	**0.961**	**0.962**	**0.995**	**0.962**	**0.962**	**0.994**	**0.959**
	DNABERT	0.773	0.783	0.848	0.774	0.793	0.860	0.544
	LS-GKM	0.785	0.778	0.883	0.837	0.726	0.869	0.577
	Enformer	0.775	0.779	0.858	0.794	0.764	0.859	0.551
**Paraxial mesoderm**	**BOM**	**0.924**	**0.927**	**0.989**	**0.930**	**0.925**	**0.988**	**0.966**
	DNABERT	0.684	0.702	0.711	0.700	0.704	0.735	0.367
	LS-GKM	0.731	0.736	0.777	0.764	0.711	0.783	0.464
	Enformer	0.761	0.763	0.843	0.800	0.730	0.836	0.525
**Pharyngeal mesoderm**	**BOM**	**0.882**	**0.884**	**0.963**	**0.871**	**0.897**	**0.966**	**0.854**
	DNABERT	0.474	0.432	0.505	0.474	0.397	0.472	-0.051
	LS-GKM	0.719	0.725	0.790	0.714	0.735	0.790	0.437
	Enformer	0.756	0.759	0.802	0.754	0.765	0.812	0.511
**Somatic mesoderm**	**BOM**	**0.960**	**0.962**	**0.996**	**0.973**	**0.951**	**0.996**	**0.985**
	DNABERT	0.472	0.000	0.566	0.000	0.000	0.548	0.000
	LS-GKM	0.815	0.815	0.909	0.867	0.768	0.887	0.637
	Enformer	0.741	0.756	0.842	0.752	0.761	0.820	0.480
**Spinal cord**	**BOM**	**0.949**	**0.952**	**0.991**	**0.921**	**0.987**	**0.991**	**0.978**
	DNABERT	0.645	0.662	0.755	0.648	0.677	0.735	0.289
	LS-GKM	0.740	0.741	0.821	0.757	0.727	0.813	0.480
	Enformer	0.751	0.796	0.860	0.687	0.946	0.862	0.540
	**BOM**	**0.877**	**0.877**	**0.917**	**0.899**	**0.856**	**0.931**	**0.931**

Cell type	Model	Accuracy	F1	auPR	Precision	Recall	auROC	MCC
**Surface ectoderm**	DNABERT	0.713	0.720	0.821	0.720	0.720	0.791	0.426
	LS-GKM	0.734	0.721	0.849	0.778	0.672	0.823	0.473
	Enformer	0.840	0.833	0.930	0.898	0.776	0.909	0.688

Supplementary Table 4. Summary of prediction statistics for random mouse E8.25 data splits
Binary BOM models was trained to predict enhancers specific to 17 mouse E8.25 cell types [3]. For every cell type, five random data splits were produced, dividing at random enhancers into training, validation and test sets. The mean accuracy, F1, auPR, precision, recall and auROC values were calculated across the 17 cell types for every random data split.

Random data split	Accuracy	F1	auPR	Precision	Recall	auROC	MCC
**#1**	0.929	0.925	0.974	0.921	0.933	0.976	0.860
**#2**	0.924	0.917	0.974	0.937	0.909	0.975	0.851
**#3**	0.919	0.914	0.965	0.914	0.915	0.970	0.837
**#4**	0.918	0.914	0.973	0.912	0.920	0.977	0.839
**#5**	0.932	0.934	0.984	0.931	0.939	0.983	0.865

Supplementary Table 5. Summary of BOM multiclass test results using different error functions
The mean value of each performance metric across E8.25 17 cell types is shown [3].

eval_metric	Recall	Precision	Accuracy	F1	auROC	auPR	MCC
mlogloss	0.882	0.985	0.995	0.926	0.999	0.988	0.927
merror	0.848	0.986	0.993	0.906	0.999	0.990	0.908

Supplementary Table 6. Summary of performance metrics of mouse E8.25 multiclass models
BOM, Basset (fine-tuned), CNN + LSTM architecture and CNNx4 + FCx2 architecture models trained to predict the cell type of mouse E8.25 enhancers. Accuracy, F1 score, auPR, precision, recall and auROC were calculated for each of the 17 cell types. 'NA' means the statistic could not be calculated because of zero denominator.

Cell type	Model	Accuracy	F1	auPR	Precision	Recall	auROC	MCC
**Allantois**	**BOM**	**0.994**	**0.903**	**0.984**	**1.000**	**0.823**	**0.999**	**0.904**
	Basset	0.968	nan	0.108	nan	0.000	0.735	0.000
	CNN + LSTM	0.968	nan	0.104	nan	0.000	0.764	0.000
	CNNx4 + FCx2	0.968	nan	0.121	0.000	0.000	0.778	-0.004
**Cardiomyocyte**	**BOM**	**0.996**	**0.971**	**0.993**	**0.994**	**0.949**	**0.999**	**0.969**
	Basset	0.930	0.065	0.221	0.545	0.034	0.784	0.124
	CNN + LSTM	0.932	0.115	0.312	0.647	0.063	0.838	0.187
	CNNx4 + FCx2	0.928	0.032	0.222	0.300	0.017	0.798	0.057
**Endothelium**	**BOM**	**0.996**	**0.985**	**0.998**	**0.983**	**0.986**	**1.000**	**0.982**
	Basset	0.941	0.786	0.858	0.828	0.748	0.954	0.753
	CNN + LSTM	0.923	0.658	0.813	0.920	0.512	0.942	0.653
	CNNx4 + FCx2	0.879	0.667	0.793	0.555	0.834	0.936	0.615
**Erythroid**	**BOM**	**0.996**	**0.973**	**0.996**	**0.966**	**0.980**	**1.000**	**0.971**
	Basset	0.919	0.205	0.352	0.520	0.127	0.840	0.229
	CNN + LSTM	0.924	0.195	0.524	0.719	0.113	0.903	0.265
	CNNx4 + FCx2	0.923	0.409	0.442	0.555	0.324	0.880	0.386
**ExE endoderm**	**BOM**	**0.998**	**0.885**	**0.972**	**1.000**	**0.793**	**1.000**	**0.889**
	Basset	0.988	nan	0.144	nan	0.000	0.888	0.000
	CNN + LSTM	0.988	nan	0.062	nan	0.000	0.789	0.000
	CNNx4 + FCx2	0.988	nan	0.040	nan	0.000	0.749	0.000
**Forebrain**	**BOM**	**0.995**	**0.934**	**0.995**	**0.989**	**0.885**	**1.000**	**0.933**
	Basset	0.960	0.108	0.249	0.857	0.058	0.813	0.216
	CNN + LSTM	0.958	nan	0.123	nan	0.000	0.764	0.000
	CNNx4 + FCx2	0.958	nan	0.095	nan	0.000	0.738	0.000
**Gut**	**BOM**	**0.995**	**0.951**	**0.993**	**0.969**	**0.933**	**1.000**	**0.948**
	Basset	0.953	0.396	0.515	0.667	0.281	0.915	0.414
	CNN + LSTM	0.946	nan	0.123	nan	0.000	0.729	0.000
	CNNx4 + FCx2	0.946	nan	0.103	nan	0.000	0.682	0.000
**Mesenchyme**	**BOM**	**0.995**	**0.935**	**0.981**	**0.979**	**0.895**	**0.999**	**0.934**
	Basset	0.961	0.284	0.389	0.655	0.181	0.877	0.331
	CNN + LSTM	0.957	nan	0.106	0.000	0.000	0.753	-0.004

Cell type	Model	Accuracy	F1	auPR	Precision	Recall	auROC	MCC
	CNNx4 + FCx2	0.958	nan	0.191	nan	0.000	0.820	0.000
**Mid/hindbrain**	**BOM**	**0.994**	**0.896**	**0.983**	**1.000**	**0.812**	**0.999**	**0.898**
	Basset	0.967	0.046	0.157	1.000	0.024	0.775	0.151
	CNN + LSTM	0.966	nan	0.088	nan	0.000	0.758	0.000
	CNNx4 + FCx2	0.966	nan	0.093	nan	0.000	0.768	0.000
**Mixed mesoderm**	**BOM**	**0.994**	**0.682**	**0.937**	**1.000**	**0.517**	**0.999**	**0.717**
	Basset	0.988	nan	0.033	nan	0.000	0.613	0.000
	CNN + LSTM	0.988	nan	0.023	nan	0.000	0.738	0.000
	CNNx4 + FCx2	0.988	nan	0.033	nan	0.000	0.741	0.000
**Neural crest**	**BOM**	0.994	0.943	0.992	0.961	0.925	**1.000**	0.940
	Basset	0.947	nan	0.171	nan	0.000	0.772	0.000
	CNN + LSTM	0.947	nan	0.142	nan	0.000	0.756	0.000
	CNNx4 + FCx2	0.928	0.304	0.246	0.315	0.293	0.838	0.266
**NMP**	**BOM**	**0.994**	**0.962**	**0.994**	**0.958**	**0.967**	**1.000**	**0.959**
	Basset	0.916	0.119	0.271	0.583	0.066	0.776	0.176
	CNN + LSTM	0.927	0.445	0.526	0.629	0.344	0.882	0.431
	CNNx4 + FCx2	0.924	0.264	0.428	0.739	0.160	0.851	0.322
**Paraxial mesoderm**	**BOM**	**0.996**	**0.968**	**0.997**	**0.975**	**0.962**	**1.000**	**0.966**
	Basset	0.939	0.105	0.284	0.750	0.057	0.816	0.195
	CNN + LSTM	0.936	nan	0.160	nan	0.000	0.743	0.000
	CNNx4 + FCx2	0.936	nan	0.132	nan	0.000	0.733	0.000
**Pharyngeal mesoderm**	**BOM**	**0.993**	**0.848**	**0.982**	**1.000**	**0.735**	**0.999**	**0.854**
	Basset	0.973	nan	0.090	nan	0.000	0.733	0.000
	CNN + LSTM	0.973	nan	0.106	nan	0.000	0.766	0.000
	CNNx4 + FCx2	0.973	nan	0.059	nan	0.000	0.723	0.000
**Somitic mesoderm**	**BOM**	**0.997**	**0.987**	**0.998**	**0.992**	**0.981**	**1.000**	**0.985**
	Basset	0.891	0.111	0.281	0.405	0.065	0.775	0.128
	CNN + LSTM	0.922	0.560	0.661	0.689	0.471	0.919	0.530
	CNNx4 + FCx2	0.902	0.229	0.415	0.692	0.137	0.820	0.279
**Spinal cord**	**BOM**	**0.996**	**0.980**	**0.999**	**0.982**	**0.978**	**1.000**	**0.978**
	Basset	0.910	0.082	0.256	0.500	0.045	0.781	0.129
	CNN + LSTM	0.917	0.290	0.392	0.627	0.188	0.848	0.313
	CNNx4 + FCx2	0.912	0.180	0.377	0.558	0.108	0.836	0.218

Cell type	Model	Accuracy	F1	auPR	Precision	Recall	auROC	MCC
**Surface ectoderm**	**BOM**	**0.994**	**0.932**	**0.995**	**0.991**	**0.88**	**1.000**	**0.931**
	Basset	0.951	0.281	0.366	0.522	0.192	0.869	0.296
	CNN + LSTM	0.950	nan	0.183	nan	0.000	0.743	0.000
	CNNx4 + FCx2	0.950	0.074	0.192	0.500	0.040	0.780	0.131

Supplementary Table 7. Flanking regions prediction statistics
Using a multiclass BOM model to predict between specific CREs of a cell type using other cell-type-specific CREs as background, we included flanking CRE regions (+-2 kb) into the prediction as a negative set based on a threshold of 0.5. Sequences were from the mouse E8.25 dataset [3].

Recall	Precision	F1	MCC	Specificity (TNR)	Acc	FPR	auROC	auPR	TP	TN	FP	FN	Cell type
0.949	0.396	0.559	0.611	**0.995**	0.995	**0.005**	0.999	0.878	166	53428	253	9	cardiom
0.906	0.025	0.049	0.14	**0.884**	0.884	**0.116**	0.972	0.678	327	96307	12667	34	endothelium
0.946	0.011	0.021	0.083	**0.713**	0.714	**0.287**	0.942	0.569	193	44191	17782	11	erythroid
0.862	0.015	0.029	0.1	**0.847**	0.847	**0.153**	0.922	0.022	25	9370	1694	4	exeEndo
0.942	0.068	0.127	0.247	**0.956**	0.956	**0.044**	0.993	0.833	98	28892	1336	6	forebrain
0.948	0.013	0.026	0.097	**0.762**	0.763	**0.238**	0.955	0.369	128	30291	9437	7	gut

Supplementary Table 8. CRE number subsample prediction statistics
We constructed successive binary models to predict cell-type specific endothelium CREs from the mouse E8.25 dataset [3]. We varied the number of randomly sampled CREs using matched numbers of cell-type-specific CREs from other cell types. These CREs were split into their respective training, validation and test sets.

N_by_class	Recall	Precision	F1	MCC	Specificity	Acc	FPR	auROC	auPR	TP	TN	FP	FN
**30**	0.714	1	0.833	0.714	1	0.833	0	0.829	0.916	5	5	0	2
**80**	0.8	0.632	0.706	0.394	0.588	0.688	0.412	0.767	0.812	12	10	7	3
**130**	0.778	0.955	0.857	0.746	0.96	0.865	0.04	0.926	0.941	21	24	1	6
**180**	0.741	0.69	0.714	0.534	0.8	0.778	0.2	0.848	0.765	20	36	9	7
**230**	0.795	0.729	0.761	0.525	0.729	0.761	0.271	0.853	0.794	35	35	13	9
**280**	0.862	0.833	0.847	0.678	0.815	0.839	0.185	0.908	0.908	50	44	10	8
**330**	0.913	0.851	0.881	0.743	0.825	0.871	0.175	0.941	0.948	63	52	11	6
**380**	0.911	0.9	0.906	0.802	0.89	0.901	0.11	0.966	0.967	72	65	8	7
**430**	0.883	0.847	0.865	0.694	0.808	0.849	0.192	0.923	0.935	83	63	15	11
**480**	0.835	0.887	0.86	0.71	0.876	0.854	0.124	0.935	0.942	86	78	11	17
**530**	0.894	0.788	0.838	0.667	0.769	0.83	0.231	0.919	0.923	93	83	25	11
**580**	0.907	0.877	0.892	0.776	0.868	0.888	0.132	0.941	0.938	107	99	15	11
**630**	0.854	0.902	0.877	0.755	0.902	0.877	0.098	0.94	0.946	111	110	12	19
**680**	0.893	0.875	0.884	0.74	0.846	0.871	0.154	0.954	0.966	133	104	19	16
**730**	0.93	0.942	0.936	0.862	0.933	0.932	0.067	0.984	0.987	146	126	9	11
**780**	0.843	0.931	0.884	0.78	0.935	0.888	0.065	0.949	0.96	134	143	10	25
**830**	0.904	0.955	0.929	0.863	0.958	0.931	0.042	0.983	0.985	150	159	7	16
**880**	0.92	0.92	0.92	0.841	0.921	0.92	0.079	0.971	0.971	161	163	14	14
**930**	0.914	0.904	0.909	0.828	0.914	0.914	0.086	0.97	0.968	160	180	17	15
**980**	0.927	0.941	0.934	0.872	0.945	0.936	0.055	0.983	0.982	177	190	11	14
**1030**	0.879	0.913	0.895	0.787	0.909	0.893	0.091	0.959	0.965	188	180	18	26
**1080**	0.947	0.934	0.94	0.875	0.928	0.938	0.072	0.984	0.985	213	192	15	12
**1130**	0.881	0.915	0.898	0.783	0.904	0.892	0.096	0.964	0.972	215	188	20	29

**1180**	0.929	0.944	0.937	0.864	0.936	0.932	0.064	0.978	0.982	236	204	14	18
**1230**	0.915	0.955	0.935	0.867	0.953	0.933	0.047	0.979	0.982	236	223	11	22
**1280**	0.943	0.935	0.939	0.875	0.932	0.938	0.068	0.979	0.982	246	234	17	15
**1330**	0.957	0.971	0.964	0.925	0.969	0.962	0.031	0.992	0.993	265	247	8	12
**1380**	0.93	0.947	0.938	0.873	0.943	0.937	0.057	0.984	0.984	267	250	15	20
**1430**	0.953	0.969	0.961	0.92	0.967	0.96	0.033	0.991	0.992	285	264	9	14
**1480**	0.944	0.941	0.942	0.882	0.938	0.941	0.062	0.984	0.987	286	271	18	17
**1530**	0.942	0.945	0.944	0.886	0.943	0.943	0.057	0.988	0.99	294	283	17	18
**1580**	0.884	0.96	0.921	0.845	0.961	0.921	0.039	0.981	0.983	290	292	12	38
**1630**	0.922	0.955	0.938	0.872	0.951	0.936	0.049	0.982	0.985	318	292	15	27
**1680**	0.946	0.971	0.958	0.914	0.969	0.957	0.031	0.992	0.992	331	312	10	19
**1730**	0.952	0.96	0.956	0.91	0.959	0.955	0.041	0.989	0.991	336	325	14	17

Supplementary Table 9. Prediction statistics for mouse E8.25 topics
A multiclass BOM model was trained to predict the topic of topic-specific mouse E8.25 CREs [3]. Mean values of accuracy, F1 score, auPR, precision, recall and auROC were calculated for the predictions of CREs specific to 93 topics.

Accuracy	F1	auPR	Precision	Recall	auROC	MCC
0.995	0.712	0.859	0.943	0.588	0.995	0.737

Supplementary Table 10. Summary of prediction statistics of mouse E8.5 CREs using models trained on mouse E8.25 CREs
Mouse E8.5 enhancers specific to 15 cell types were scored using binary BOM models trained on mouse E8.25 enhancers to distinguish similar cell types. The models were trained on enhancers trimmed to their central 500bp and E8.5 enhancers were trimmed in a similar way. Mean values of accuracy, F1, auPR, precision, recall and auROC values were calculated across the predictions produced by the 15 models.

Accuracy	F1	auPR	Precision	Recall	auROC	MCC
0.759	0.692	0.847	0.831	0.606	0.852	0.532

Supplementary Table 11. Super enhancers (SE) vs. non-super enhancer (classical) prediction statistics

Cell type	CRE type	Recall	Precision	F1	MCC	Specificity	Acc	auPR	auROC	TP	TN	FP	FN
Cardiomyocyte	SE	0.929	0.975	0.951	0.87	0.957	0.938	0.991	0.984	39	22	1	3
Cardiomyocyte	classical	0.955	0.969	0.962	0.929	0.973	0.965	0.994	0.995	127	145	4	6
Forebrain	SE	1	1	1	1	1	1	1	1	7	15	0	0
Forebrain	classical	0.938	0.989	0.963	0.919	0.986	0.959	0.998	0.997	91	73	1	6

Supplementary Table 12. Summary of prediction statistics for different motif detection thresholds
Binary BOM models were trained to distinguish enhancers specific to 17 mouse E8.25 cell types and 6 human cell lines. We trained the models on datasets produced with different motif detection thresholds (q-value <= 0.1, q-value <= 0.3 and q-value <= 0.5). Mean values of accuracy, F1 score, auPR precision, recall and auROC were calculated for each dataset and every motif detection threshold (N = 17 and 6 mouse E8.25 cell types and human cell lines, respectively).

Dataset	q-value threshold	Accuracy	F1	auPR	Precision	Recall	auROC	MCC
**Mouse E8.25**	<= 0.1	0.882	0.883	0.956	0.891	0.880	0.952	0.766
	<= 0.3	0.936	0.937	0.983	0.939	0.936	0.982	0.873
	<= 0.5	0.934	0.933	0.984	0.933	0.937	0.984	0.869
**Human cell lines**	<= 0.1	0.688	0.670	0.774	0.707	0.666	0.769	0.392
	<= 0.3	0.869	0.872	0.948	0.848	0.902	0.950	0.743
	<= 0.5	0.923	0.926	0.978	0.893	0.962	0.980	0.850

Supplementary Table 13. Summary of prediction statistics of BOM models trained on overlapping and non-overlapping TF binding motifs counts
Binary BOM models were trained to predict enhancers specific to each of the 17 mouse E8.25 cell types. Mean accuracy, F1 score, auPR, precision, recall and auROC values were calculated across the 17 models.

Model motifs	Accuracy	F1	auPR	Precision	Recall	auROC	MCC
**Non-overlapping**	0.602	0.532	0.686	0.680	0.454	0.664	0.235
**overlapping**	0.934	0.933	0.984	0.933	0.937	0.984	0.869

Supplementary Table 14. Prediction statistics for human cell lines
Binary BOM models were trained to predict cell line specific CREs against a background composed of CREs from the other cell lines. We used a total of 6 cell lines (Gm12878, Hela-S3, Huvec, H1-hESC, HepG2 and K562). We calculated accuracy, F1 score, auPR, precision, recall and auROC across the 6 models. We used q-value <= 0.5 as motif detection threshold.

Accuracy	F1	auPR	Precision	Recall	auROC	MCC
0.923	0.926	0.978	0.893	0.962	0.980	0.850

Supplemental Table 15. Summary of prediction statistics of human hematopoiesis enhancers
Binary BOM models were trained to predict enhancers specific to 22 cell types. Mean accuracy, F1 score, auPR, precision, recall and auROC values were calculated across the 22 data sets.

Accuracy	F1	auPR	Precision	Recall	auROC	MCC
0.897	0.901	0.949	0.873	0.934	0.958	0.799

Supplemental Table 16. Summary of prediction statistics of zebrafish enhancers
Binary BOM models were trained to predict enhancers specific to 11 adult zebrafish tissues. Mean values of accuracy, F1, auPR, precision, recall and auROC across the 11 test sets are shown.

Accuracy	F1	auPR	Precision	Recall	auROC	MCC
0.960	0.961	0.991	0.941	0.983	0.992	0.921

Supplementary Table 17. *A. thaliana* enhancers prediction statistics
Prediction statistics for the multiclass classification of A. thaliana root cell types.

Cell type	Accuracy	F1	auPR	Precision	Recall	auROC	MCC
**Endodermis 2**	0.910	0.718	0.878	0.875	0.609	0.960	0.682
**Endodermis 3**	0.877	0.667	0.835	0.938	0.517	0.934	0.639
**Xylem (Stele 1)**	0.934	0.818	0.869	0.947	0.720	0.940	0.790
**Precursor of cortex/endodermis**	0.885	0.837	0.948	0.878	0.800	0.969	0.751

Supplementary Table 18. Human fetal enhancers prediction statistics
Binary BOM models were trained to predict human fetal enhancers specific to cardiomyocytes and erythroblast cells. The values of accuracy, F1 score, auPR, precision, recall and auROC are shown for each model.

Cell type	Accuracy	F1	auPR	Precision	Recall	auROC	MCC
**Cardiomyocytes**	0.949	0.952	0.974	0.915	0.992	0.981	0.901
**Erythroblasts**	0.873	0.851	0.931	0.952	0.769	0.923	0.755

Supplementary Table 19. Cross-species prediction statistics of cell type specific CREs
Binary BOM models were trained to distinguish human or mouse cell-type specific CREs cardiomyocyte ('CM') and erythroblasts ('ER'). The models were tested in CREs from a similar cell type of the other species. The mean accuracy, F1 score, auPR, precision, recall and auROC values are shown in each case.

Species (model)	Species (test data)	Cell type	Accur.	F1	auPR	Prec.	Recall	auROC	MCC
**Mouse**	Human	CM	0.755	0.723	0.835	0.821	0.645	0.850	0.520
		ER	0.706	0.688	0.845	0.759	0.629	0.813	0.421
**Human**	Mouse	CM	0.711	0.652	0.833	0.823	0.540	0.814	0.450
		ER	0.763	0.743	0.805	0.809	0.688	0.817	0.532

Supplementary Table 20. Mouse adult heart enhancers prediction statistics
Prediction statistics of BOM multiclass model trained to predict mouse adult heart cell type-specific CREs. The model was trained to distinguish CREs specific to common human-mouse cell types (**Methods**).

Cell type	Accuracy	F1	auPR	Precision	Recall	auROC	MCC
**Cardiomyocyte**	0.990	0.989	1.000	0.981	0.998	1.000	0.979
**Endothelial**	0.984	0.937	0.984	0.931	0.944	0.997	0.928
**Fibroblast**	0.990	0.976	0.998	0.959	0.994	1.000	0.970
**Lymphocyte**	0.988	0.910	0.974	0.952	0.872	0.997	0.905
**Macrophage**	0.986	0.827	0.954	0.958	0.727	0.997	0.828
**Nervous**	0.988	0.830	0.954	0.966	0.728	0.998	0.833
**Smooth Muscle**	0.992	0.815	0.964	0.985	0.695	0.998	0.824

Supplementary Table 21. Human adult heart enhancers prediction statistics
Prediction statistics of BOM multiclass model trained to predict human adult heart cell type-specific CREs. Only human-mouse shared cell types were included (**Methods**).

Cell type	Accuracy	F1	auPR	Precision	Recall	auROC	MCC
**Cardiomyocyte**	0.997	0.995	1.000	0.992	0.998	1	0.993
**Endothelial**	0.995	0.977	0.998	0.984	0.969	1	0.974
**Fibroblast**	0.998	0.995	1.000	0.992	0.997	1	0.993
**Lymphocyte**	0.997	0.609	0.968	1.000	0.438	1	0.660
**Macrophage**	0.996	0.989	1.000	0.985	0.993	1	0.987
**Nervous**	0.996	0.930	0.993	0.989	0.879	1	0.930
**Smooth Muscle**	0.994	0.980	0.999	0.984	0.975	1	0.976

Supplementary Table 22. Cross-species prediction of adult heart cell type-specific CREs
Performance statistics for the cross-species prediction of cell type-specific adult heart CREs. CM = cardiomyocyte, EC = endothelial cells, FB = fibroblast, LC = lymphocyte, MAC = macrophage, NER = nervous cells and SM = smooth muscle.

Species (model)	Species (test data)	Cell type	Accur.	F1	auPR	Prec.	Recall	auROC	MCC
**Mouse**	Human	CM	0.896	0.836	0.955	0.949	0.747	0.974	0.773
		EC	0.872	0.555	0.628	0.418	0.823	0.930	0.528
		FB	0.868	0.670	0.755	0.710	0.635	0.910	0.589
		LC	0.886	0.097	0.203	0.052	0.748	0.904	0.177
		MAC	0.896	0.612	0.777	0.846	0.480	0.933	0.587
		NER	0.975	0.227	0.385	0.817	0.132	0.879	0.322
		SM	0.872	0.108	0.370	0.641	0.059	0.775	0.167

**Human**	Mouse	CM	0.832	0.849	0.936	0.765	0.953	0.939	0.684
		EC	0.852	0.495	0.497	0.463	0.532	0.861	0.410
		FB	0.821	0.206	0.608	0.838	0.117	0.853	0.271
		LC	0.935	0.115	0.449	0.933	0.062	0.851	0.230
		MAC	0.952	0.385	0.354	0.440	0.342	0.894	0.363
		NER	0.957	0.156	0.143	0.246	0.114	0.787	0.148
		SM	0.933	0.151	0.092	0.105	0.272	0.786	0.139

Supplementary Table 23. Human adult heart cell prediction statistics.
Statistics for the prediction performance of human cells using SHAP scores from the multiclass model trained to classify mouse cell type-specific CREs (**Methods**).

Cell type	Accuracy	F1	Precision	Recall	MCC
**Cardiomyocyte**	0.946	0.928	0.996	0.869	0.891
**Fibroblast**	0.932	0.904	0.935	0.876	0.853
**Macrophage**	0.971	0.823	0.859	0.790	0.808
**Endothelial**	0.954	0.700	0.592	0.856	0.689
**Smooth Muscle**	0.940	0.550	0.724	0.444	0.538
**Adipocyte**	0.999	NA	NA	0.000	NA
**Nervous**	0.972	0.099	0.053	0.719	0.191

Supplementary Table 24. Summary of prediction performance metrics for BOM models trained using different tree depth values
We used the mouse E8.25 cell type-specific enhancers dataset to evaluate the effect of the maximum decision tree depth in the classification performance (XGBoost parameter "max_depth"). BOM binary models were trained for each of the 17 cell types using a maximum tree depth of 6 (default), 8, 10 and 12. The mean values of accuracy, F1 score, auPR, precision, recall and auROC across the 17 cell types are shown.

Decision trees depth	Accuracy	F1	auPR	Precision	Recall	auROC	MCC
**6 (default)**	0.934	0.933	0.984	0.933	0.937	0.984	0.869
**8**	0.941	0.939	0.986	0.940	0.942	0.986	0.882
**10**	0.938	0.937	0.986	0.933	0.945	0.986	0.877
**12**	0.938	0.937	0.985	0.935	0.943	0.985	0.877

Supplementary Table 25. Summary of performance of DNN multiclass models on test data

All tasks were executed on a compute node with 12 CPUs and the best performance is highlighted in bold. *_without Grid Search: Model was trained using pre-selected hyperparameters, without performing a grid search. *_with Grid Search: Model was trained using the optimal hyperparameters identified through a grid search. *_fine-tuned: The model was fine-tuned on a pre-trained model, utilizing pre-trained weights and training only the final output layer for specificity.

**CNNx3 + FCx2** (Basset) training/fine-tuning	Model Size (number of parameters)	Hyperparameter selection		Performance on test set			Training log		
		lr	batch_size	AVE_auc	AVE_auPR	MCC	Wall_Time	CPU_Time	Epoch
CNNx3 + FCx2_without grid search	3,993,317	0.002	128	0.625	0.108	0.1132	12m50s	2h0m50s	29
CNNx3 + FCx2_optimal hyperparameters	3,993,317	0.0026	128	0.654	0.124	0.145	12m44s	1h56m50s	28
**Basset_fine-tuned**	17,017	0.0080	64	**0.807**	**0.279**	**0.322**	5m33s	46m48s	48

**CNN + LSTM** (DeepMel**)** training/fine-tuning	Model Size (number of parameters)	Hyperparameter selection		Performance on test set			Training log		
		lr	batch_size	AVE_auc	AVE_auPR	MCC	Wall_Time	CPU_Time	Epoch
**CNN + LSTM_without grid search**	3,440,401	0.001	128	**0.802**	**0.262**	**0.337**	7m32s	42m42s	30
CNN + LSTM_optimal hyperparameter	3,440,401	0.0012	128	0.789	0.245	0.320	7m36s	47m41s	32
DeepMel_fine-tuned	4,369	0.0084	64	0.660	0.132	0.098	15m39s	1h36m38s	48

**CNNx4 + FCx2** training/fine-tuning	Model Size (number of parameters)	Hyperparameter selection		Performance on test set			Training log		
		lr	batch_size	AVE_auc	AVE_auPR	MCC	Wall_Time	CPU_Time	Epoch
CNNx4 + FCx2_without grid search	1,120,113	0.002	128	0.706	0.154	0.168	3m10s	22m50s	11
**CNNx4 + FCx2_optimal hyperparameter**	1,120,113	0.0083	64	**0.792**	**0.234**	**0.283**	3m10s	22m48s	11

Supplementary References

1. Grant, C. E., Bailey, T. L. & Noble, W. S. FIMO: scanning for occurrences of a given motif. *Bioinforma. Oxf. Engl.* **27**, 1017–1018 (2011).
2. Chen, T. & Guestrin, C. XGBoost: A Scalable Tree Boosting System. in *Proceedings of the 22nd ACM SIGKDD International Conference on Knowledge Discovery and Data Mining* 785–794 (New York, USA, 2016).
3. Pijuan-Sala, B. *et al.* Single-cell chromatin accessibility maps reveal regulatory programs driving early mouse organogenesis. *Nat. Cell Biol.* **22**, 487–497 (2020).
4. Bruse, N. & Heeringen, S. J. van. GimmeMotifs: an analysis framework for transcription factor motif analysis. 474403 Preprint at https://doi.org/10.1101/474403 (2018).
5. Akerberg, B. N. *et al.* A reference map of murine cardiac transcription factor chromatin occupancy identifies dynamic and conserved enhancers. *Nat. Commun.* **10**, 4907 (2019).
6. Roadmap Epigenomics Consortium *et al.* Integrative analysis of 111 reference human epigenomes. *Nature* **518**, 317–330 (2015).
7. Madsen, J. G. S. *et al.* Integrated analysis of motif activity and gene expression changes of transcription factors. *Genome Res.* **28**, 243–255 (2018).
8. Balwierz, P. J. *et al.* ISMARA: automated modeling of genomic signals as a democracy of regulatory motifs. *Genome Res.* **24**, 869–884 (2014).
9. Lee, D. LS-GKM: a new gkm-SVM for large-scale datasets. *Bioinformatics* **32**, 2196–2198 (2016).
10. Schep, A. N., Wu, B., Buenrostro, J. D. & Greenleaf, W. J. chromVAR: inferring transcription-factor-associated accessibility from single-cell epigenomic data. *Nat. Methods* **14**, 975–978 (2017).
