# Supplementary material for: Motif-based models accurately predict cell type-specific distal regulatory elements
Source: Nat Commun. 2025 Nov 24;16:10370. doi: 10.1038/s41467-025-65362-2 (PMC12644898; doi:10.1038/s41467-025-65362-2)
Supplement: Supplementary file 2 — Description of Additional Supplementary Files [file 41467_2025_65362_MOESM2_ESM.pdf]

Description of Additional Supplementary Files:

Supplementary Dataset 1: Motif IDs and consensus sequences implanted in muscle enhancer for luciferase assay

Supplementary Dataset 2: Synthetic Regulatory Element (SRE) sequences used in luciferase assay

Supplementary Dataset 3: Synthetic Regulatory Element (SRE) activity values
